# Tooth resorption—Part 2: A clinical classification

**DOI:** 10.1111/edt.12762

**Published:** 2022-05-23

**Authors:** Paul V. Abbott, Shaul Lin

**Affiliations:** ^1^ UWA Dental School The University of Western Australia Crawley Western Australia Australia; ^2^ Department of Endodontic and Dental Trauma Rambam Health Care Campus Haifa Israel; ^3^ The Ruth and Bruce Rappaport Faculty of Medicine Technion – Israel Institute of Technology Haifa Israel; ^4^ The Israeli National Center for Trauma & Emergency Medicine Research, The Gertner Institute for Epidemiology and Health Policy Research Sheba Medical Canter Tel Hashomer Israel

**Keywords:** classification, inflammatory resorption, invasive resorption, replacement resorption, tooth resorption

## Abstract

Tooth resorption is either a physiological or a pathological process resulting in loss of dentin and/or cementum. It may also be associated with bone loss. Currently there is no universal classification for the different types of tooth resorption. This lack of a universal classification leads to both confusion amongst practitioners and poor understanding of the resorptive processes occurring in teeth which can result in incorrect/inappropriate diagnoses and mis‐management. When developing a classification of diseases and/or conditions that occur within the body, several criteria should be followed to ensure a useful classification. The classification should not only include pathological conditions but also physiological conditions. Since tooth resorption can be either pathological or physiological, a classification of tooth resorption should include both of these categories. Any classification of diseases should be possible to use clinically, meaningful, useful, clear and universal. It should enable easy storage, retrieval and analysis of health information for evidenced‐based decision‐making. It should also be possible to share and compare data and information between different institutions, settings and countries. A classification of tooth resorption should be developed by combining anatomical, physiological and pathological approaches. For some types of resorption, the aetiological approach should also be incorporated. A classification of tooth resorption that uses simple, relevant and appropriate terminology based on the nature and location of the resorptive process occurring in teeth is proposed. There are two broad categories of internal and external tooth resorption which are sub‐divided into three types of internal tooth resorption (surface, inflammatory, replacement) and eight types of external tooth resorption (surface, inflammatory, replacement, invasive, pressure, orthodontic, physiological, idiopathic). The clinician's understanding, diagnosis and management of tooth resorption can be facilitated by using this simple classification which should ideally be used universally by the entire dental profession to ensure clarity and to avoid confusion.

## 1 INTRODUCTION

Resorption can be defined as either a physiological or a pathological condition that results in a loss of substance from a tissue.^1^ In dentistry, resorption may result in the loss of dentin, cementum and/or bone.^2^ “Tooth resorption” rather than “root resorption” is a more appropriate term because resorption can involve the root and/or the crown of the tooth.

There are several types of resorption that affect the teeth. Each type will have one or more specific aetiologies. Each type of resorption will also have its own specific pathogenesis. Hence, a comprehensive understanding of the aetiology and pathogenesis of each type of resorption is essential in order to be able to diagnose the condition and then to manage it with an appropriate treatment regime or, in some cases, to monitor its progress until a definitive treatment (such as root canal treatment, extraction, etc.) becomes necessary.

**FIGURE 1:**
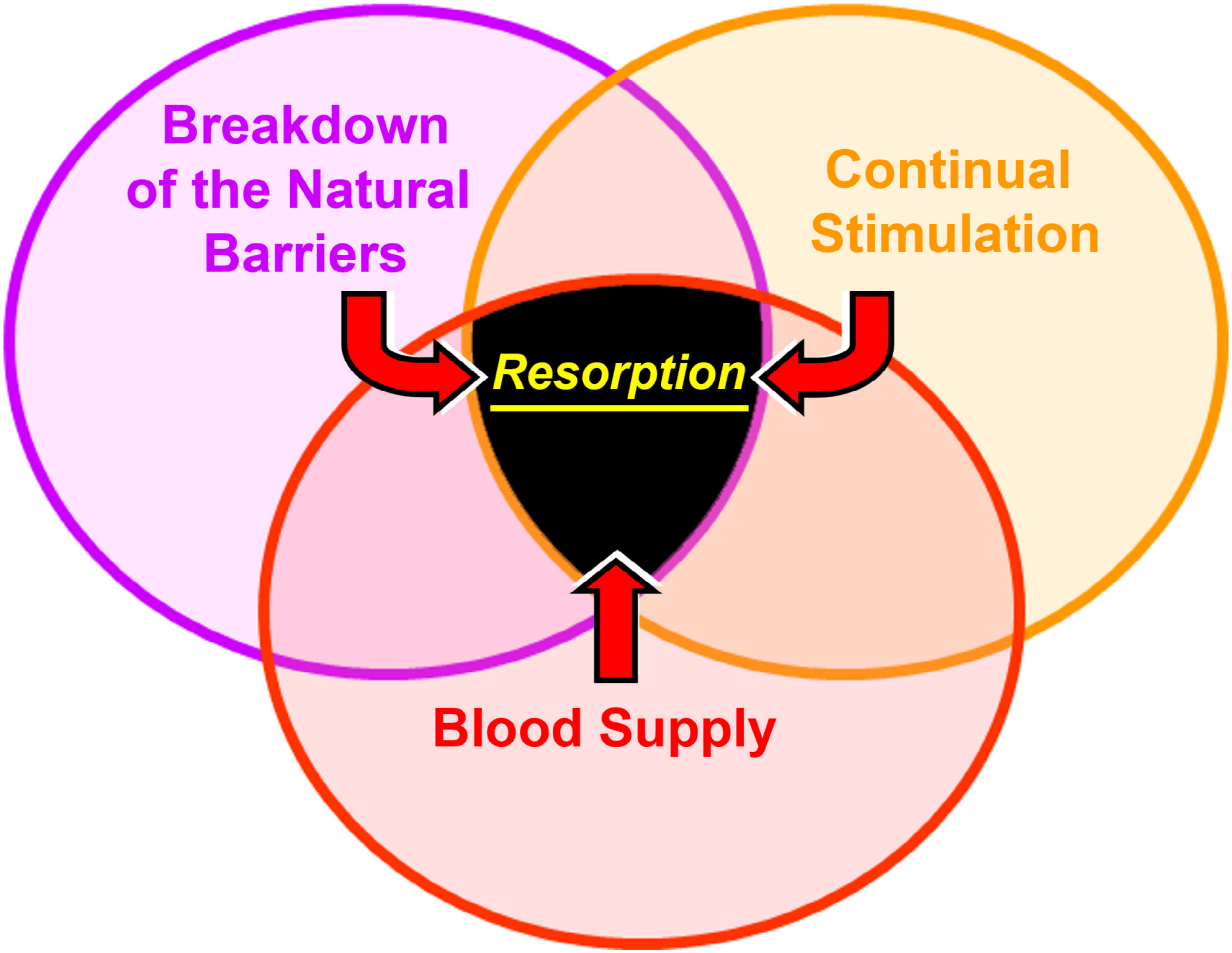
The “Resorption Triad” showing the three general requirements for resorption to occur

There are several requirements for tooth resorption to occur. Each type of resorption involves its own specific requirements, as detailed below. However, there are three general requirements that can be called the “Resorption Triad” (Figure 1): (a) breakdown of the natural barriers in the tissues, (b) a continuous stimulating factor, and (c) a viable blood supply for the clastic cells. All three components of the above triad need to be present for resorption to occur. The external natural barriers are the periodontal ligament (PDL) and the cementum while the internal natural barriers are the odontoblasts and predentin. These barriers prevent clastic cells from adhering to, or resorbing, the unmineralized matrix of the cementum and dentin.^3^ Most likely a combination of the physical presence of these barriers as well as their biochemical compositions are responsible for their barrier actions.^4^ Stimulating factors for resorption may be the presence of bacteria in the root canal system, necrosis of the PDL after traumatic injuries, developmental defects, an adjacent impacted tooth, an adjacent cyst or tumor, etc.^4^, ^5^

The above three components of the triad should be considered when formulating a management plan for any tooth that has resorption—for example, in some cases, treatment may be possible to preserve the tissues (e.g. external inflammatory resorption), but if the resorption is already present then this contributing factor can no longer be managed or altered by treatment. In some cases, the stimulating factor can be removed (e.g. bacteria within the root canal system for both internal and external inflammatory resorption), whilst other cases may be managed by removing the blood supply (e.g. by performing root canal treatment for internal inflammatory resorption).^6^, ^7^

In order to diagnose the specific type of resorption that is present, a classification of the different types of resorption is necessary. Many classifications have been used in the dental literature but with little consistency between them and with many different terms for the same type of resorption.^8^ A narrative review in 2018^9^ identified 15 different classifications that were published between 1970 and 2016, plus the authors proposed an additional classification resulting in a total of at least 16 classifications in the dental literature. Hence, it is no surprise that there is considerable confusion amongst dental practitioners regarding tooth resorption. Consequently, the aims of this paper are to: (1) discuss the purposes and requirements of a classification of diseases/conditions; (2) discuss how to develop a classification of diseases/conditions; (3) outline a classification of tooth resorption based on the pathological and/or physiological processes that occur with each type of resorption; and (4) provide a definition and a brief outline of each type of resorption in this classification.

## 2 PURPOSES AND REQUIREMENTS OF A CLASSIFICATION

The first stages of managing any disease or condition are to take a thorough history from the patient, followed by a clinical and radiographic examination in order to make a diagnosis. It is imperative that an accurate diagnosis be obtained as this will determine the options for managing the particular disease or condition that is present.^7^ In medicine and dentistry, there can be various diseases or conditions affecting a single tissue or organ. They may have some similarities plus some differences—the differences are critical as they will usually mean that the management and/or treatment must be different.

The terms used for the diagnosis of any disease or condition are basically a few words that summarise a particular combination of symptoms reported by the patient, clinical signs, radiographic observations and the results of various diagnostic tests.^7^ Some conditions may have similar clinical presentations and therefore it is important that clinicians differentiate between them in order to provide the appropriate management for the condition.^7^ It is also presumed that the diagnosing clinician and any other clinician involved in treating the patient will know the meaning of the terms used in the diagnosis.

If only a few words are used to summarise a disease or condition, a classification is required for all related or similar diseases and conditions of any tissue or organ. Examples of this in dentistry include the classifications of pulp and peri‐radicular conditions, periodontal conditions and the various types of tooth resorption. When developing a classification, it is necessary to understand the physiological or pathological changes that have occurred in the tissues and how these develop and progress over time. In addition to the above, classifications of dental and medical conditions should satisfy the following criteria^7^, ^10^—they should be:

1. *Possible to use* in a clinical setting—that is, the diagnosis can be determined based on the information gained from the patient's symptoms, clinical signs, tests, radiographs, etc.
2. *Meaningful* **—**that is, the various conditions reflect what is happening, or has already happened, in the involved tissues of the body.
3. *Useful*—that is, the diagnosis directs the clinician to the management options for the particular condition or disease.
4. *Clear*—that is, the diagnosis and terminology used can be understood by all relevant clinicians.
5. *Universal*—that is, the classification should be used throughout the world to standardise terminology and allow effective communication among clinicians, educators, students and researchers.

In keeping with the “Universal” criterion listed above, the World Health Organisation^11^ has stated that classifications of diseases are important to enable easy storage, retrieval and analysis of health information for evidenced‐based decision‐making, to facilitate sharing and comparing of health information between hospitals, regions, settings and countries, and to allow data comparisons in the same location across different time periods.

Unfortunately, the various classifications of tooth resorption in the literature have not followed these basic principles of classification. There is also little consistency between the many classifications and many different terms used for the same type of resorption.^8^, ^9^ Hence, it is timely and essential to develop a new classification of tooth resorption that follows the above principles.

## 3 DEVELOPING A CLASSIFICATION

According to Scarpelli et al.,^12^ a classification of diseases and/or conditions should be based on one or more of the overarching approaches listed below. Any single disease or condition may fall within one or more of these types of classifications. The suggested approaches are to classify the diseases/conditions according to the relevant: (1) Topography—by bodily region or system; (2) Anatomy—by organ or tissue; (3) Physiology—by function or effect; (4) Pathology—by the nature of the disease processes; (5) Aetiology—by the cause(s) of the diseases/conditions; (6) Juristic—according to the legal circumstances of death, especially when it is sudden; (7) Epidemiology—by incidence, distribution and control of the disorders; or (8) Statistics—by analysis of the incidence and prevalence rate of the diseases/conditions.^12^

Since tooth resorption must involve teeth, the first approach (topography) is largely irrelevant and unnecessary. The last three approaches (juristic, epidemiology and statistics) are not relevant to clinical dental practice and can be ignored when considering a classification for tooth resorption.

In nine of the 16 classifications identified in a narrative review by Aidos et al.,^9^ an attempt was made to use the aetiology approach. Examples of these include the classifications by Fuss et al.,^13^ Lindskog et al.,^14^ Heithersay,^15^ Santos et al.,^16^ Darcy and Qualtrough,^17^ and Sak et al.^18^ Other classifications have combined the aetiology approach with trauma,^4^ with the location and type of resorption,^19^ or they have specified the aetiology as trauma only.^20^

The classifications based on aetiology (with or without location, type, etc.) are difficult to use clinically. They can also be somewhat confusing because the aetiology is not always obvious (e.g., external invasive resorption) or there may be several causes (e.g., both external and internal inflammatory resorption may be caused by trauma as well as by infection). Hence, it is difficult to have a simple (or single) classification for these resorptive conditions solely based on aetiology. In addition, some types of resorption have specific causes and therefore they cannot be easily grouped with other types. For instance, both pressure resorption and orthodontic resorption have been categorized as “trauma‐induced”^14^, ^15^ but they are not related to accidental trauma which is what is generally implied when discussing trauma to the teeth. They are also quite different to other types of resorption that may be induced by (accidental) trauma, such as external replacement resorption. Notwithstanding the above, the aetiological approach can be used in conjunction with other approaches (see below) to differentiate between similar types of pathological resorptive processes which have different (and specific) aetiologies plus where the clinical management is different.

Using terms that include the word “related” (e.g., infection‐related resorption, ankylosis‐related resorption) is another approach that is essentially following the aetiology approach of classification.^21^ However, this approach is inappropriate because it does not truly indicate the aetiology, but rather only a related part of the aetiology. Furthermore, it does not indicate the pathological process involved. These types of resorption may have other related factors as well as other possible causes. For example, external inflammatory resorption (also recently called external infection‐related resorption) requires an infected root canal system—this can be related to trauma, or it may be related to caries or a restoration that has broken down to allow bacteria to enter the root canal system. Likewise, external ankylosis‐related resorption (external replacement resorption) may be related to trauma but ankylosis can also be related to other types of resorption—such as external invasive resorption or external inflammatory resorption where repair occurs without PDL between the tooth and the bone. Furthermore, similar terminology using “‐related” cannot be easily applied to other types of resorption. Hence, this approach has several flaws and it is inconsistently applied to the various types of resorption, so it should be avoided.

A combination of the second, third and fourth types of approaches to classifications listed above (anatomy, physiology and pathology, respectively), can, and should, be used to develop a classification for tooth resorption. The second approach (anatomy) is important because tooth resorption involves one or more of the tooth tissues (i.e., pulp, dentin and/or cementum) and it may also involve the adjacent bone. The specific tissue(s) involved can be included in the definition of each type of resorption (as outlined in the proposed classification below where each type of resorption is described). As discussed above, the aetiological approach can also be used in conjunction with these three approaches to differentiate between similar types of pathological resorptive processes which have different (and specific) aetiologies plus where the clinical management is different.

The location of the resorption within the tooth should not be part of the terminology used in the classification. An exception can be considered if the particular type of resorption always occurs in the same location (e.g., external apical inflammatory resorption, external lateral inflammatory resorption). However, if the particular type of resorption varies in its location, then the terminology used should not include the location (e.g., external invasive resorption, often called “cervical resorption” or various other terms that include the word “cervical”). The location of all types of tooth resorption is an important aspect to record in the patient's clinical records as this directs the clinician to appropriate management. Hence, the location could be considered simply as part of the clinical and radiographic findings in a similar manner to the diagnosis and recording of dental caries (e.g., mesial caries) or other diseases/conditions.

The physiology approach, should be applied to one type of tooth resorption since it is physiological in nature (see “physiological resorption”, described below). All other types of tooth resorption are pathological and consequently the fourth approach, pathology, should also be used for the development of a classification of the different types of tooth resorption. This latter approach is also the most important one due to the pathological nature of most types of tooth resorption.

Another important aspect of a classification is to have consistency in the terminology used. Unfortunately, this has not been the case in some of the existing classifications of tooth resorption. For example, replacement resorption, where tooth substance is lost and replaced by bone, can occur either externally or internally.^22^, ^23^ Hence, the same term (that is, “replacement resorption”) should be used as the pathological process (resorption) and the outcome (replacement by bone) are the same. In order to distinguish between the two types, the descriptors “internal” and “external” should be added.

## 4 THE CLASSIFICATION OF TOOTH RESORPTION

As outlined above, the following classification is based mainly on the anatomical, physiological and pathological processes involved in the resorption, with some also incorporating the aetiology approach. Eleven different types of resorption in two broad categories can be identified with these approaches (Table 1). The aim of the remainder of this paper is to propose a classification and to briefly describe the aetiology plus the clinical and radiographic features of each type of resorption to facilitate the diagnosis of each type of resorption. The general principles of managing each type of resorption will also be outlined. The purpose of this classification and the general outline is not to review the literature in detail but to help clinicians understand the types of resorption and to guide them towards more consistent diagnosis and management by using a simplified classification system that is mainly based on the pathological and/or physiological processes that occur with each type of resorption, plus its anatomical location.

**TABLE 1:** The 11 different types of tooth resorption based on the specific tissues involved and the pathological or physiological processes of each type of resorption

Internal tooth resorption	External tooth resorption
Internal surface resorption	External surface resorption
2 Internal inflammatory resorption	2 External Inflammatory Resorption (a) Apical (b) Lateral
3 Internal Replacement Resorption	3 External Replacement Resorption (a) Transient (b) Progressive
	4 External Invasive Resorption
	5 External Pressure Resorption
	6 Orthodontic Resorption
	7 Physiological Resorption
	8 Idiopathic Resorption

Since tooth resorption can begin either within the tooth or on the external surface of the root, the different types of tooth resorption can be divided into two broad categories, namely: (a) internal tooth resorption and (b) external tooth resorption.

*Internal tooth resorption* begins within the pulp or in the dentin of the root canal walls. It progresses outwards towards the cementum and, if not treated, it can ultimately result in communication with the PDL and surrounding bone. There are three types of internal tooth resorption—surface, inflammatory and replacement (Table 1).

*External tooth resorption* commences within the cementum and/or dentin (when there is no cementum present at the site of initiation of the resorption) and progresses inwards towards the dental pulp. If not treated, it can reach the pulp, resulting in communication between the pulp and the surrounding bone. There are eight types of external tooth resorption—surface, inflammatory, replacement, invasive, pressure, orthodontic, physiologic and idiopathic (Table 1).

## 5 INTERNAL TOOTH RESORPTION

### 5.1 Internal surface resorption

**FIGURE 2:**
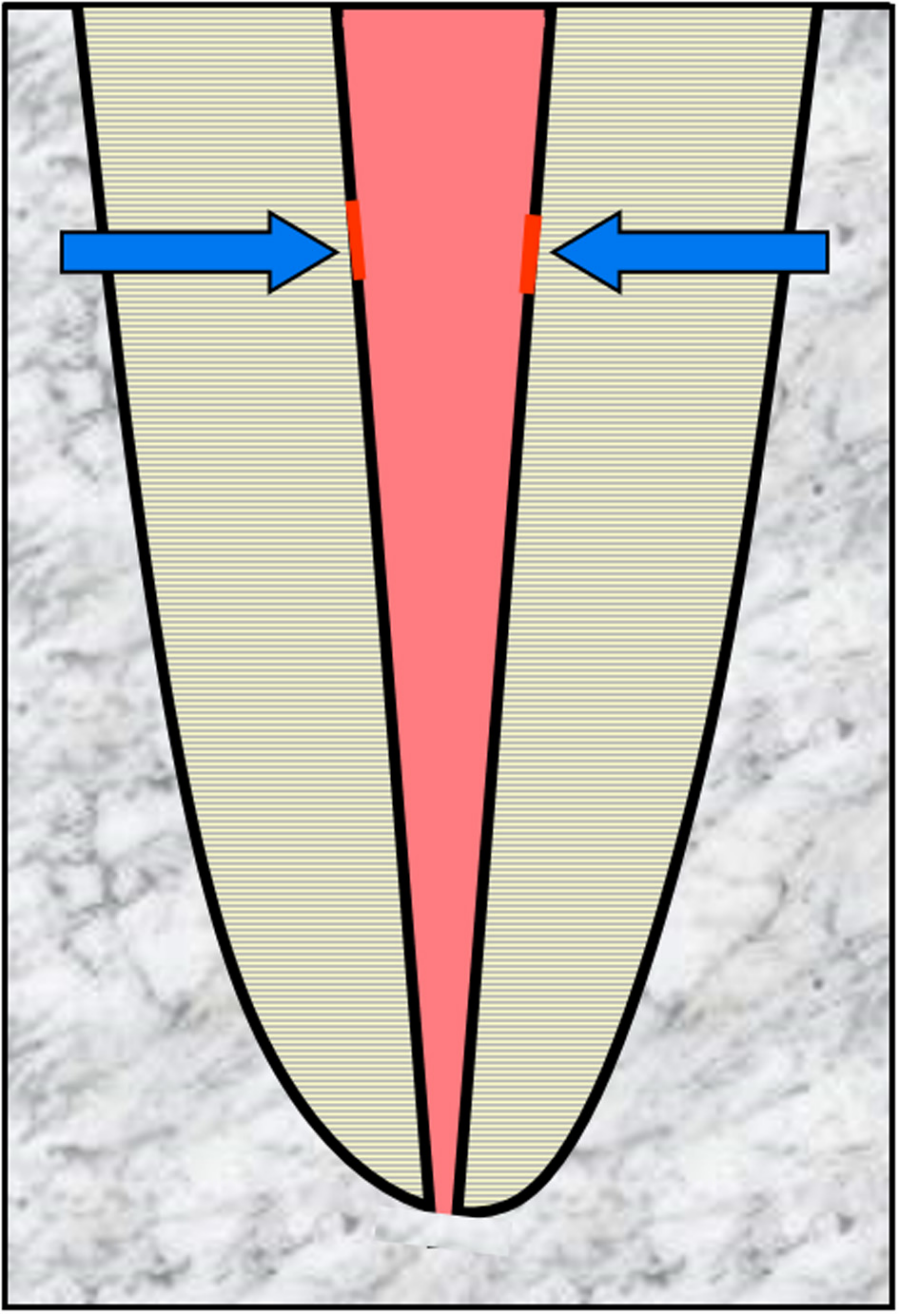
Schematic diagram demonstrating Internal Surface Resorption—arrows indicate minor areas of resorption of the root canal walls

Internal surface resorption is a rare condition that is defined as minor areas of resorption of the dentin walls of the root canal (Figure 2). It is believed to be a transient and self‐limiting process. The term “surface” is used to designate this type of resorption as it indicates the minor and shallow nature of the resorption.

The aetiology is not well understood but it appears to occur when there has been minor irritation to the pulp and where that irritation is not ongoing. The irritation may be associated with trauma to a tooth^24^, ^25^ or external bleaching of a tooth.^26^ It may also occur prior to the pulp becoming necrotic and infected as a result of caries, cracks or restoration breakdown. In the latter situation, it may be a precursor to internal inflammatory resorption because the stimulating factor (i.e. bacteria) will normally be present over a long period of time.

Histologically, the pulp adjacent to the dentin is devoid of odontoblasts and predentin. Inflammatory cells such as neutrophils and macrophages are attracted to the denuded surface and dentinoclasts can be seen in resorption lacunae. The damaged or exposed dentin/predentin only maintains resorptive activity for 2–3 weeks, provided the stimulating factor does not persist.^24^, ^26^, ^27^, ^28^

Clinically, this type of resorption cannot be detected because it occurs on the walls of the root canal. It is also not evident radiographically because the resorptive defects are very minor or shallow and there will be no symptoms or clinical signs.^29^ Hence, it is unlikely that clinicians will be able to diagnose the presence of internal surface resorption. Nevertheless, it is important to understand that this condition may be present and that it may lead to internal inflammatory resorption.^24^, ^25^, ^26^, ^27^, ^28^

There is no indication for any management of this type of resorption since it is usually self‐limiting but in cases where internal surface resorption continues (e.g. when associated with caries), it will progress and become internal inflammatory resorption which can then be diagnosed and managed as outlined below.

### 5.2 Internal inflammatory resorption

**FIGURE 3:**
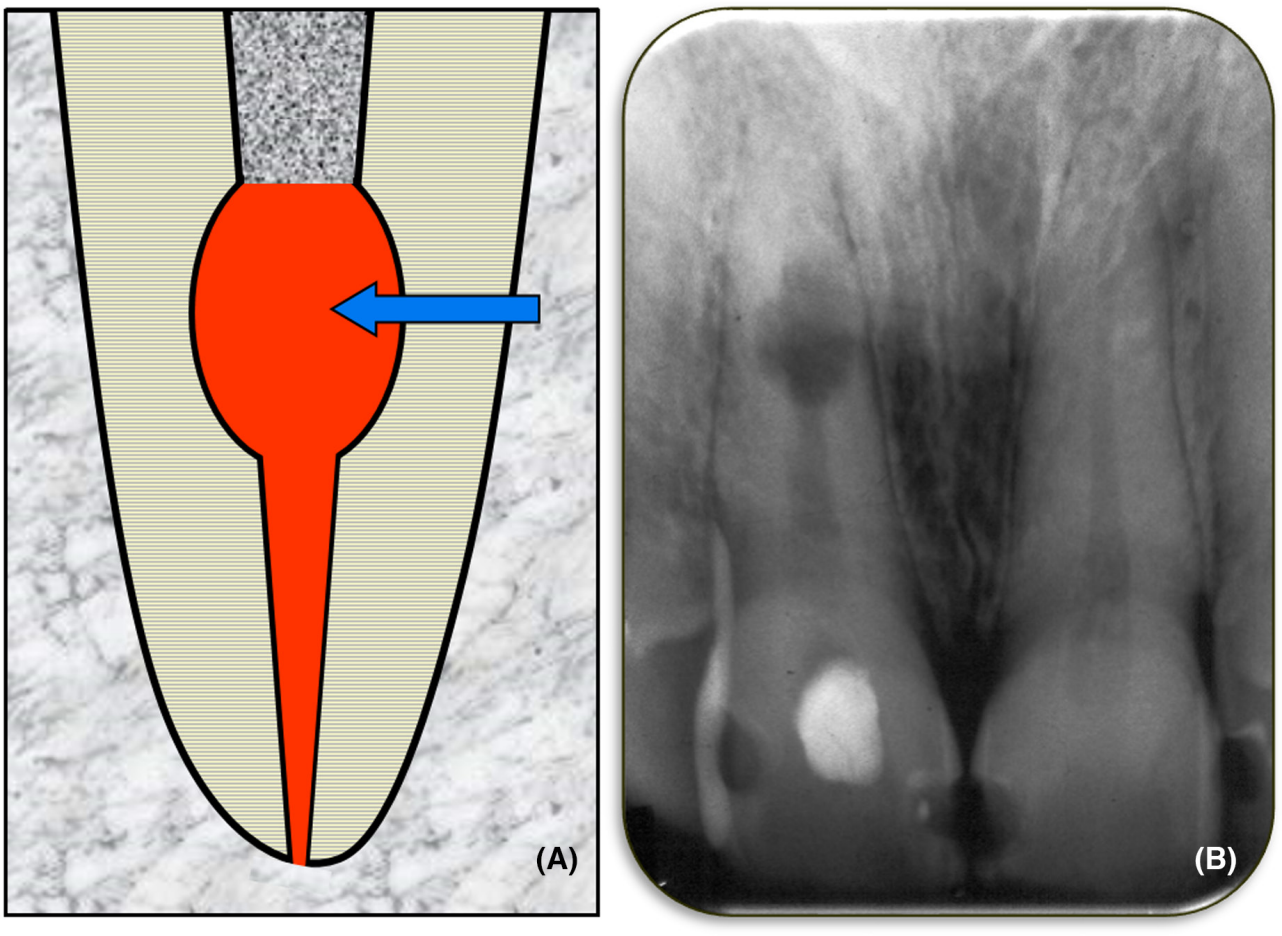
Internal Inflammatory Resorption. (A) Schematic diagram; (B) Periapical radiograph of tooth 11 with an area of internal inflammatory resorption in the mid‐root region

Internal inflammatory resorption is defined as an inflammatory process within a section of the pulp/root canal that results in loss of dentin commencing at the root canal wall and progressing towards the cementum (Figure 3). The term “inflammatory” is used to designate this type of resorption since it is an inflammatory process that leads to the loss of dentin.

Internal inflammatory resorption occurs when there is necrotic and infected pulp tissue coronal to the resorptive defect and in the dentin tubules. The pulp will not be normal with a lack of odontoblasts and predentin.^30^ The pulp will be replaced by periodontal‐like connective tissue although a histological study was unable to determine whether this tissue had replaced the pulp before or after the resorption commenced.^30^ When the resorption is active, there will be inflamed pulp tissue in the root canal apical to the resorptive defect and the resorptive defect will contain dentinoclasts and granulation tissue.^28^ However, as time progresses, the bacteria in the coronal part of the root canal system will move progressively towards the apical end of the tooth root in the same manner as in any tooth with an infected root canal system. As the bacteria progress apically, the remaining pulp will necrose and the root canal system will become pulpless and infected. Once the bacteria and the “necrotic front” have passed through the resorptive defect, there will no longer be a viable blood supply within the defect so the dentinoclasts will not survive and the resorption will stop. Subsequently, apical periodontitis will develop as it does with all teeth that have infected root canal systems.

The aetiology of internal inflammatory resorption is often linked to trauma but the majority of cases that have been reported in the literature have had only a history of caries,^25^, ^30^ a combination of caries and trauma,^30^ or a combination of caries and periodontal disease.^25^ Since this type of resorption only occurs when the coronal part of the pulp is necrotic and infected, the role of bacteria is clear and therefore it is not surprising that caries is often a factor.

Clinically, the presentation will vary depending on the stage of the resorptive process. In the early and active stages of this resorption, there are usually no symptoms or clinical signs.^29^ Since there are no symptoms, patients are not likely to visit a dentist, although occasionally teeth with active internal inflammatory resorption may develop symptoms associated with the pulpitis in the apical portion of the root canal. However, most patients with this resorption only present when the resoprtion is no longer active and they have symptoms associated with the apical periodontitis that develops when the entire root canal system becomes pulpless and infected.^29^ Such symptoms may indicate primary or secondary acute apical periodontitis, a primary or secondary acute apical abscess, or facial cellulitis.^29^, ^31^ In some cases, a clinical sign such as a draining sinus—indicating a chronic apical abscess—may be the first indication of the problem.

Radiographically, internal inflammatory resorption has been described as having a characteristic oval‐shaped increase in the size of the pulp chamber^20^, ^28^, ^32^ but the resorptive defect can be any shape or size. Horizontal tube shift radiographs and careful observation of the presence or absence of the outline of the pulp space can be used to differentiate this resorption from the various types of external resorption. Computed tomography scans can help differentiate, but they are not usually required.

The management of internal inflammatory resorption usually involves root canal treatment^25^ which could include the use of medicaments such as an antibiotic‐corticosteroid compound (e.g. Ledermix paste—OzDent Pty Ltd), if the resorption is active, followed by calcium hydroxide.^3^, ^6^ The corticosteroid component (i.e., triamcinolone) is an excellent anti‐inflammatory agent that also inhibits the clastic (resorbing) cells. Tetracycline antibiotics work in two ways ‐ one by inhibiting the bacteria causing the resorption and the other by inhibiting the clastic cells.^3^, ^6^ Calcium hydroxide should be used in both active and non‐active cases of internal inflammatory resorption as it also has several modes of action ‐ namely anti‐bacterial action, anti‐resorption action, and it helps dissolve any necrotic tissue that may be trapped in the resorptive defect.^33^

### 5.3 Internal replacement resorption

**FIGURE 4:**
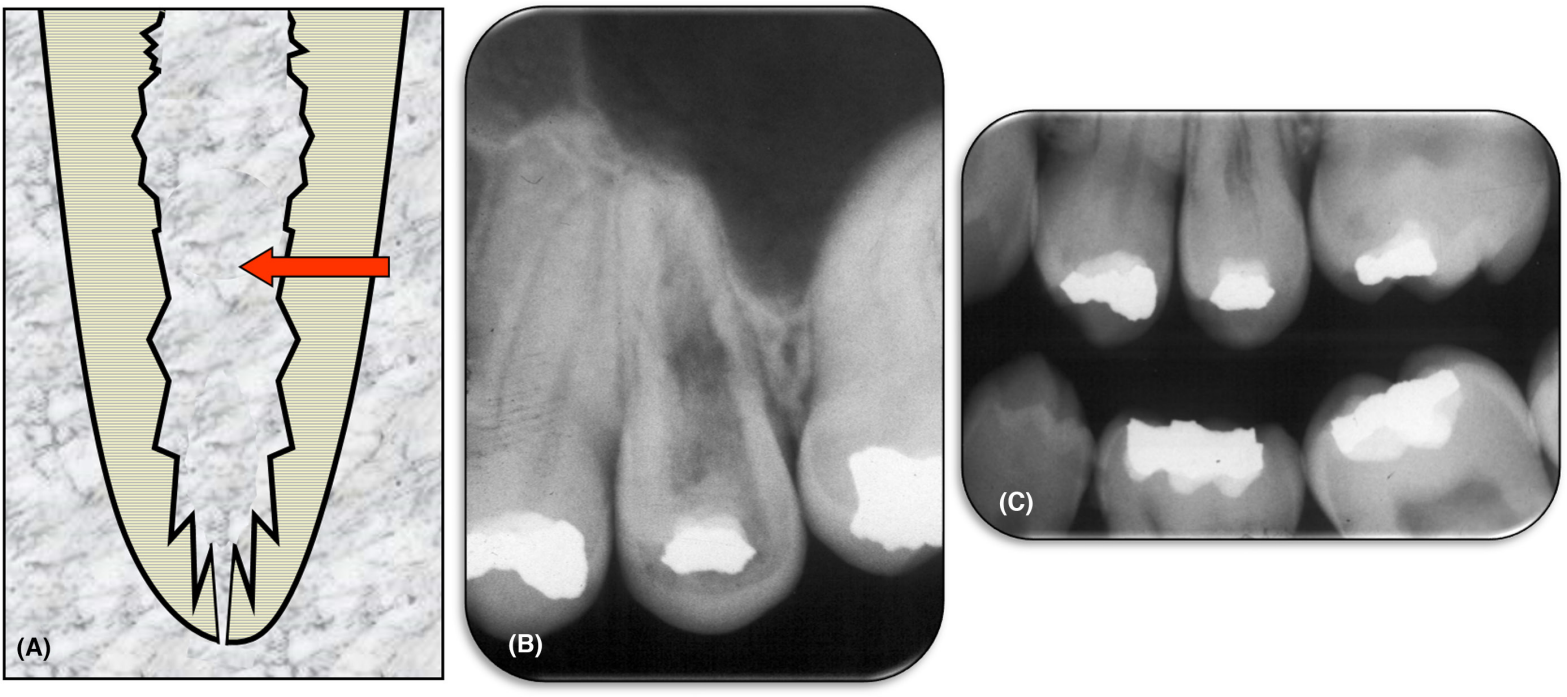
Internal Replacement Resorption. (A) Schematic diagram; (B) Periapical radiograph of tooth 25 with extensive internal replacement resorption throughout the entire tooth; (C) A bitewing radiograph taken 3 years earlier showing the pulp chamber and coronal portion of the root of the same tooth. The irregular shape and appearance of the pulp space suggests that the internal replacement resorption had likely already commenced. A comparison of these two radiographs indicates that over the 3‐year period, the resorptive process continued and bone‐like tissue replaced the lost dentin throughout the length of the root and extending into the crown of the tooth

Internal replacement resorption is a very rare condition which is defined as the process where the pulp and dentin are replaced by bone. Internal replacement resorption begins within the pulp/root canal and on the root canal walls, and it progresses towards the cementum (Figure 4). The term “replacement” is used because it describes the nature of this type of resorption.

The aetiology of internal replacement resorption is thought to be trauma or some form of insult to the pulp.^24^, ^28^ It may manifest shortly after, or years after, the trauma or insult. Hence, many patients do not recall any specific event, or they do not recall the details. In addition, this type of resorption is very rare, with many dentists unaware of its existence, so there is no definitive data available for analysis. It is possible that this type of resorption may follow internal surface resorption where there has not been a bacterial‐related cause of the surface resorption.

Clinically, the affected tooth will usually appear normal but it may be discolored or have a slight pink hue, depending on the position and the degree of progression of the resorptive process. The slight pink color only occurs if the resorptive process has extended into the pulp chamber within the crown. The patient will usually not have any symptoms associated with the tooth which may or may not respond to pulp sensibility tests. The tooth may have some ankylosis with reduced mobility and/or the typical ankylotic percussion sound. This type of resorption is often an incidental finding during a radiographic examination.^29^

Radiographically, there is an irregular enlargement of the pulp space which appears bone‐like and extends into the dentin towards the cementum. The bone that has replaced the tooth structure will resemble cancellous bone.^29^

Management of internal replacement resorption is limited to observation with regular clinical and radiographic examinations, and eventual extraction of the tooth. Root canal treatment might be feasible if the resorption is diagnosed at a very early stage but early diagnosis is uncommon due to the lack of symptoms and clinical signs.

## 6 EXTERNAL TOOTH RESORPTION

### 6.1 External surface resorption

**FIGURE 5:**
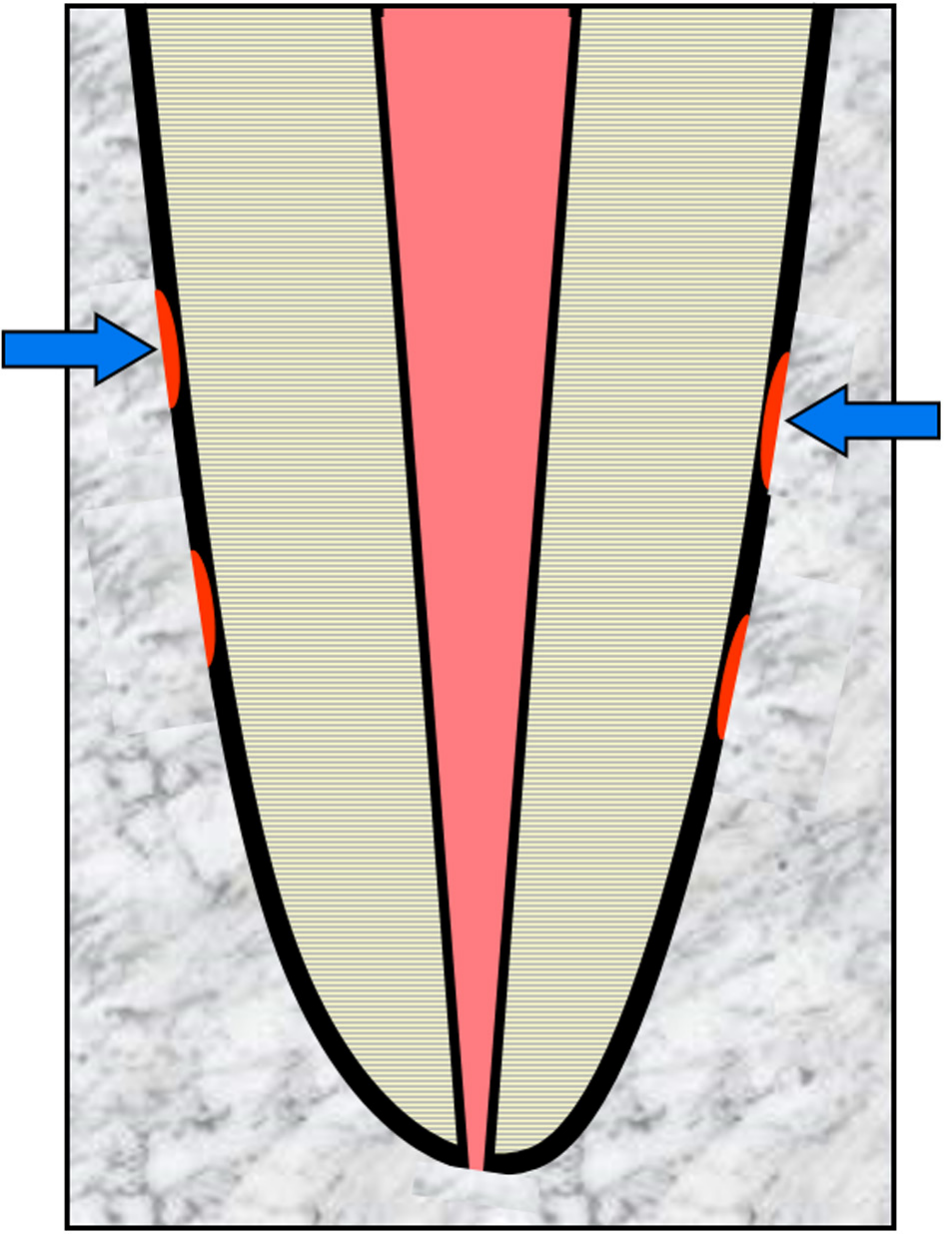
Schematic diagram demonstrating External Surface Resorption – arrows indicate minor areas of resorption of the cementum

External surface resorption is defined as small areas of resorption of the cementum. Occasionally it may extend into the dentin^17^ (Figure 5). The term “surface” is used to describe this type of resorption because it usually only involves the external surface of the tooth.

External surface resorption is a self‐limiting process which is usually caused by a localized injury to the involved part of the cementum and/or PDL. Since most injuries are only a momentary event, and as long as the tooth has not been contaminated with bacteria, the resorptive process will not persist. External surface resorption can be considered to be part of the healing response following a minor injury.

Clinically, external surface resorption will not be evident as there will be no symptoms or clinical signs.^29^ A history of recent trauma to the tooth could suggest the possibility of this type of resorption being present for a brief period of a few weeks after the injury.

Radiographically, external surface resorption will not usually be evident as a result of the shallow nature of the resorptive defect. Typically, it is revealed only during histological examination.^34^

There is no indication for any management of this type of resorption since it is usually self‐limiting, provided the irritation is of a short duration and the tooth has not been contaminated by bacteria. This is fortunate because this type of resorption is unlikely to be diagnosed. In cases where the root canal system has become infected during the injury, the resorption will progress to become external inflammatory resorption which can then be diagnosed and managed as outlined below.

### 6.2 External inflammatory resorption

**FIGURE 6:**
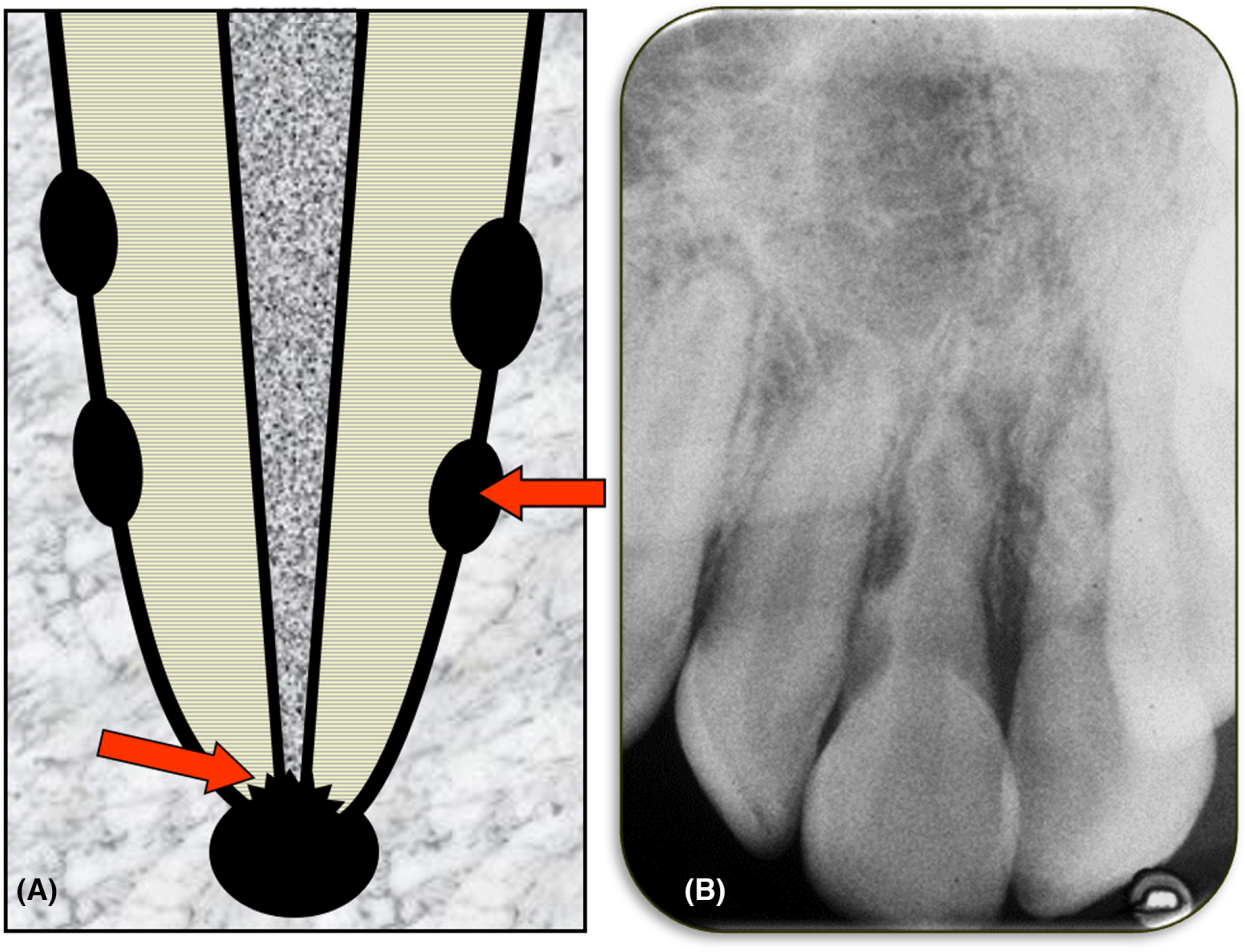
External Inflammatory Resorption. (A) Schematic diagram; (B) Periapical radiograph of tooth 11 with several sites of external inflammatory resorption, especially on the distal surface

External inflammatory resorption (Figure 6) occurs when the tooth has an infected root canal system and there has been either damage to the external surface of the tooth root (i.e., damage to the cementum and/or PDL) during a traumatic injury such as luxation or avulsion, or there is a pathway of communication between the root canal system and the peri‐radicular tissues (such as, the apical foramen or a lateral canal foramen) and bacteria and/or their endotoxins are able to escape into the peri‐radicular tissues.^3^, ^6^ The bacteria and/or their endotoxins induce an inflammatory reaction, or if inflammation is already present following an injury, the escape of bacteria and/or their endotoxins will exacerbate the inflammation. If the infected root canal system is not treated, then the result will be continued inflammation and clastic cells will be activated to resorb the tooth and the adjacent bone.^3^, ^6^ This condition has been called infection‐related resorption^21^ even though it is not solely due to the infection in the canal as there needs to be either damage to the root surface or another pathway for bacteria and/or their endotoxins to escape. The reaction in the tissues is inflammation which continues as long as the tooth remains untreated. Hence, the term “inflammatory” is appropriate since it is a more accurate description of what happens in the tissues at the site of the resorption.

**FIGURE 7:**
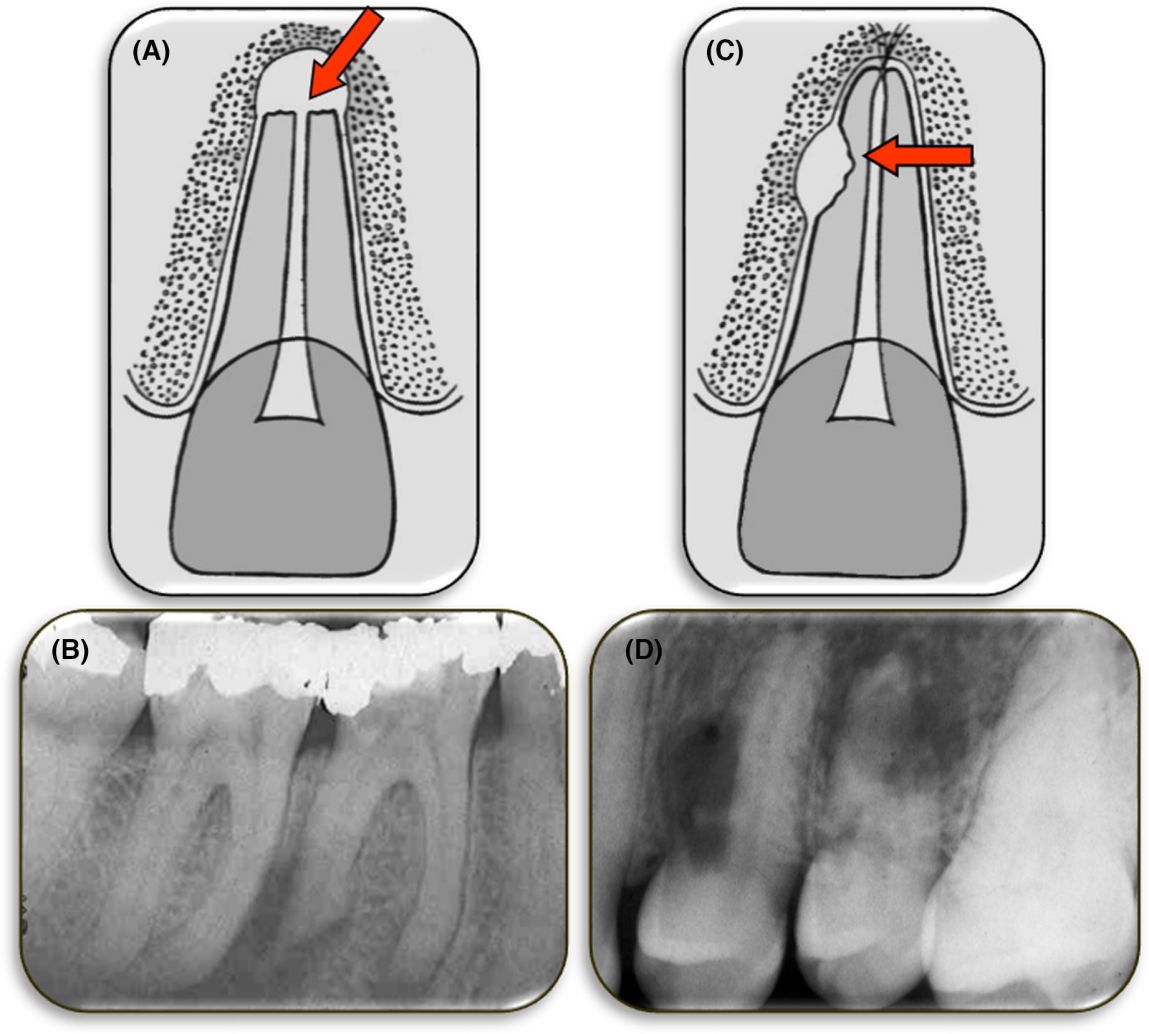
Two types of External Inflammatory Resorption. (A) Diagram demonstrating external apical inflammatory resorption; (B) Periapical radiograph of tooth 36 with extensive external apical inflammatory resorption of both the mesial and distal roots; (C) Diagram demonstrating external lateral inflammatory resorption; (D) Radiograph of teeth 24 and 25 with extensive external apical inflammatory resorption of their roots

External inflammatory resorption can occur in two locations—either at the apex of the tooth root or at any other location along the length of the tooth root (Figure 7).^6^ These are designated as either: (a) “External Apical Inflammatory Resorption”, or (b) “External Lateral Inflammatory Resorption”.

If external inflammatory resorption occurs after trauma, then it is most commonly lateral inflammatory resorption, although it could also occur apically.^6^ If external inflammatory resorption is associated with a long‐standing infected root canal system, it typically occurs apically but it may occur laterally if associated with an infected lateral canal and its foramen.^6^ The main reason to differentiate between these locations is related to the treatment (see below).

One of the causes of external inflammatory resorption is a traumatic injury such as luxation or avulsion of a tooth where the pulp's blood supply is severed and does not recover following repositioning of the tooth. In addition, bacteria must contaminate the root canal system either during the injury or subsequently for this resorption to occur. Another cause is a long‐standing infected root canal system which in turn may be caused by caries, cracks, fractures, breakdown of a restoration, or by very deep periodontal pockets involving a lateral or apical foramen of the tooth.^6^

Clinically, many scenarios are possible and the symptoms and signs will depend on the aetiology. If the tooth has undergone trauma, then there will be a history of the injury. There will be no direct symptoms associated with this type of resorption but the patient may have symptoms associated with the injury, and this will depend on the timing of the examination with respect to when the injury occurred.^29^

If the tooth has had a long‐standing infected root canal system that is causing external inflammatory resorption, then the patient may have no, or only occasional mild, symptoms if there is chronic apical periodontitis. However, there may be pain when biting and on percussion if there is acute apical periodontitis. If there is an acute apical abscess, there will also be swelling. Some cases may present with a draining sinus which indicates a chronic apical abscess. In all these situations, the pulp will not respond to pulp sensibility tests and the cause (caries, cracks, restoration breaking down, etc.) of the infected root canal system should be obvious. The tooth will have a necrotic and infected pulp, a pulpless and infected root canal system, or a root‐filled and infected root canal system if there has been previous root canal treatment and the root canal system has become infected again.^6^, ^10^, ^29^, ^31^

Radiographically, this type of resorption has a “classic” appearance which is described as a radiolucency within the tooth root due to loss of external tooth substance together with a radiolucency of the bone adjacent to the resorptive defect. The lamina durra will not be evident at the site of the resorption. If there is external lateral inflammatory resorption, the radiolucency within the tooth root has been described as a “bowl‐shaped” radiolucency but this is not always the case as it may have any shape and can vary in size, depending on the extent of the resorption. If there is external apical inflammatory resorption, then the root apex will have an irregular appearance (it may appear “moth‐eaten”) because of the loss of tooth structure. It will also have an open apical foramen and a periapical radiolucency.^29^

Management of external inflammatory resorption depends on the presentation and aetiology. Comprehensive reviews of this type of resorption and strategies for its management have been presented^3^, ^6^, ^35^ with two management approaches which have been called the “preventive approach” and the “interceptive approach”.

The preventive approach is advocated following traumatic injuries to teeth where the external root surface and/or the PDL have been damaged plus where pulp necrosis and infection are predictable.^3^, ^6^, ^35^ There are six injuries that meet these criteria. Four are in teeth with fully developed (mature) roots—namely, avulsion, intrusion, lateral luxation with a concurrent crown fracture, and extrusion with a concurrent crown fracture. The other two are in teeth with incompletely developed (immature) roots—namely, avulsion with a concurrent crown fracture, and intrusion with a concurrent crown fracture.^3^, ^6^, ^35^

The preventive approach is based on two principles. The first principle is to use systemic antibiotics immediately after the teeth have been repositioned and stabilized. The second principle is to commence root canal treatment immediately after stabilization and medicate the canal with a corticosteroid‐antibiotic (CS‐AB) paste (e.g., Ledermix paste). The CS‐AB mixture is used because of the properties of the two active components which can reduce inflammation, inhibit clastic cells and kill bacteria.^6^, ^35^, ^36^, ^37^ After 3 months of the CS‐AB medicament (including 2–3 changes of the medicament to ensure that an adequate amount is always present in the canal), calcium hydroxide [Ca(OH)_2_] is introduced into the canal in order to ensure all bacteria have been destroyed and to encourage hard tissue repair in incompletely developed teeth. The 3 months of the CS‐AB medicament is advocated in order to allow time for the PDL to repair before using the Ca(OH)_2_.^6^, ^35^ Early use of Ca(OH)_2_ should be avoided because it leads to ankylosis and subsequent external replacement resorption since it favours osseous repair rather than PDL repair by fibroblasts.^37^, ^38^, ^39^, ^40^

The interceptive approach is recommended when external inflammatory resorption is already present. The general approach is similar to the preventive approach except systemic antibiotics are not required and they will not arrest the resorption.^41^ Root canal treatment with the use of the same CS‐AB paste as an initial medicament for 3 months will usually arrest the resorption. Then, root canal medication with Ca(OH)_2_ is recommended to stimulate repair of the hard tissue that has been lost through the resorptive process—that is, the adjacent bone and the cementum.^6^, ^35^

### 6.3 External replacement resorption

**FIGURE 8:**
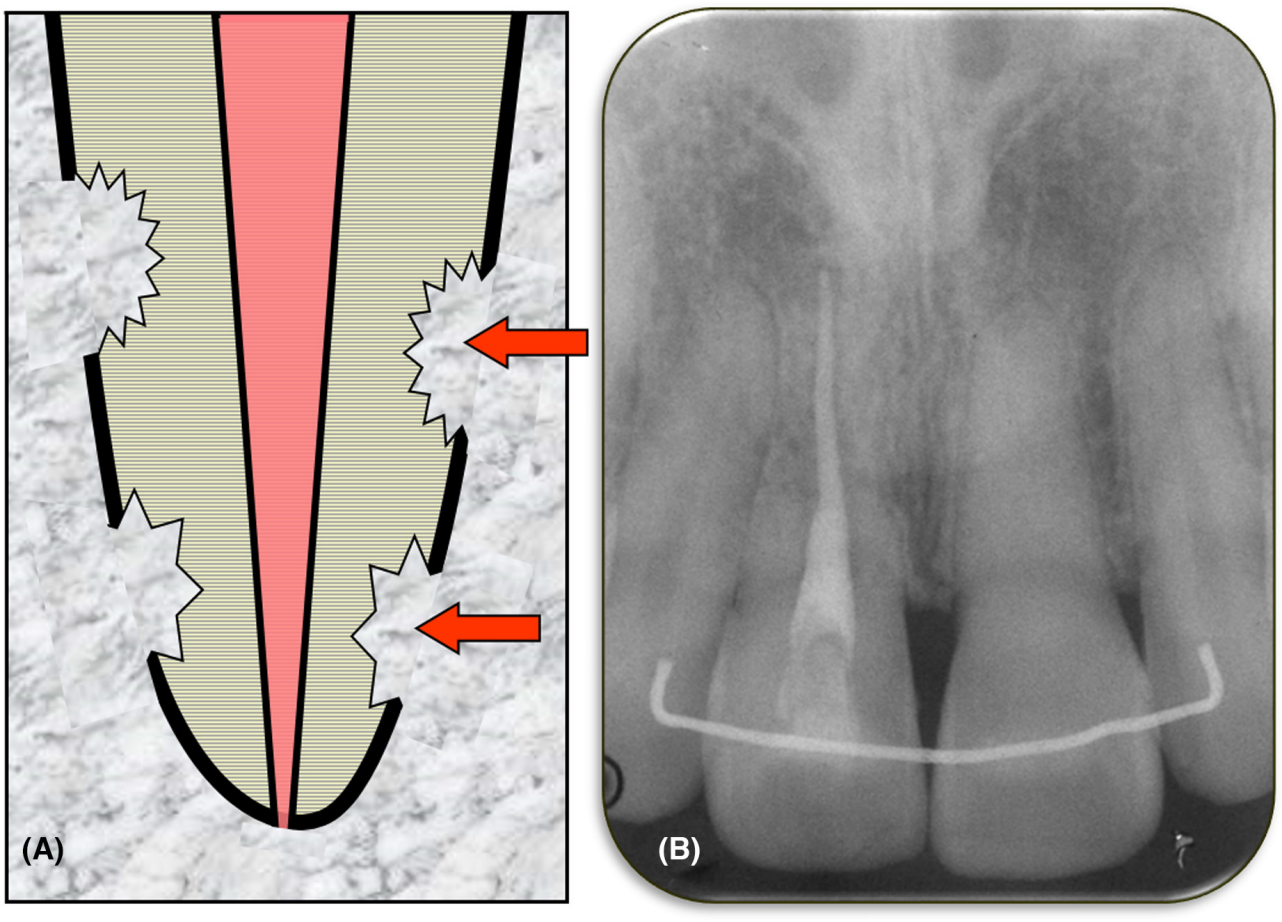
External Replacement Resorption. (A) Schematic diagram; (B) Periapical radiograph of tooth 11 with extensive external replacement resorption, especially of the apical two‐thirds of the root

External replacement resorption is the process where cementum and dentin are resorbed and replaced by bone (Figure 8). The term “replacement” is used as it describes the nature of this resorption.

**FIGURE 9:**
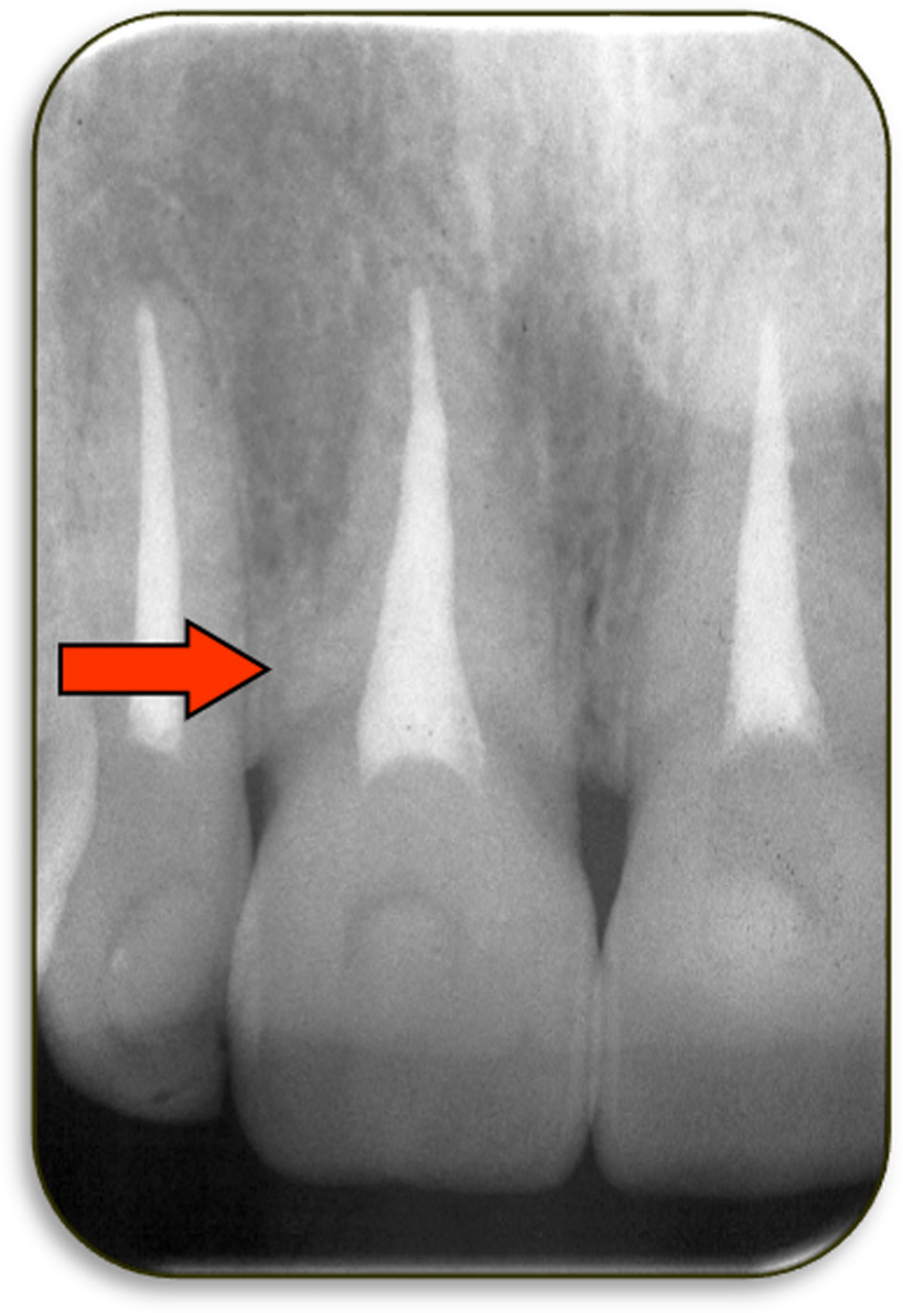
Periapical radiograph of tooth 11 which had been avulsed and replanted within a few minutes. This radiograph was taken 3 years after the injury and it shows an area of ankylosis (i.e. no periodontal ligament) but there is no external replacement resorption at this stage. The arrow indicates the region of ankylosis on the distal aspect of the cervical third of the root. This tooth also had reduced mobility and the typical percussion sound of ankylosis

In discussing this type of resorption it is important to also discuss ankylosis as this term has been incorrectly and interchangeably used to describe external replacement resorption.^2^ Another term that has been commonly used in recent years is “ankylosis‐related resorption”.^21^ However, as discussed above, no pathological condition should be named according to its relationship with another condition, especially if it is not exclusively related to it. Ankylosis is defined as the loss of PDL such that the bone and tooth root (i.e. cementum and/or dentin) are in direct contact with each other and they appear to be fused together.^2^ Ankylosis can occur without any resorption (Figure 9)—such as when it initially begins. Eventually, and with varying time frames according to the aetiology of the ankylosis, it will lead to external replacement resorption because the protective function of the PDL is not present at the site of the ankylosis. However, ankylosis can also be associated with other types of external resorption—such as following external inflammatory resorption, external invasive resorption, external pressure resorption, orthodontic resorption, physiological resorption, and idiopathic resorption. The development of ankylosis depends on the nature of the healing response that occurs following the treatment of these types of resorption, or in some cases it may be part of the progressive nature of the resorption (e.g. external invasive resorption^42^, ^43^, ^44^, ^45^). Hence, the use of the term “ankylosis‐related resorption” is inappropriate. Likewise, ankylosis should be distinguished from external replacement resorption. The term “ankylosis and replacement resorption” is more correct, and more appropriate, because this type of resorption will always have ankylosis. However, when “external replacement resorption” is used, it can be assumed that there is also ankylosis.

External replacement resorption typically occurs following an injury to the PDL and/or external root surface (i.e. the cementum).^21^, ^22^, ^23^, ^35^, ^46^ It is very common after severe luxation injuries such as avulsion and intrusion. It can also occur after lateral luxation – especially in the apical part of the root where the root scrapes across the bony ledge created when the labial alveolar cortical plate fractures, and also on the palatal aspect of the coronal third of the root which is forced against the alveolar socket wall, resulting in comminution of the bone and crushing of the PDL and cementum. It is less likely to occur after extrusion since there is less damage to the root surface with this injury. However, the PDL is damaged so it is still possible that external replacement resorption may occur.

Following injury to the PDL and/or cementum, the PDL cells may necrose which leads to ankylosis because the osseous healing response dominates.^34^ The tooth then loses the protection provided by the PDL which allows clastic cells from the adjacent bone to resorb the cementum and dentin. This will be followed by osteoblasts forming bone in the area of resorption. This process is usually progressive, eventually leading to resorption of the entire root and loss of the tooth.^22^, ^23^, ^46^ However, it might also be transient in some cases if the damage was not severe and only involves a small area of the PDL and root. In these cases, the PDL repairs through the action of fibroblasts in the adjacent PDL. This latter situation has been termed “external surface resorption” because of the repair that occurs.^8^ However, it is not necessarily the same as outlined above for external surface resorption because such resorption is not associated with ankylosis or any replacement of the tooth substance by bone whereas with true transient replacement resorption, there will be some replacement of tooth by bone followed by the repair. Clinically, there will be signs of ankylosis (such as reduced mobility, different percussion sound and feeling) in the early (or active) stage, followed by a return to normal mobility, percussion sound and feeling once the resorption has ceased and the PDL has repaired.^29^ Following the explanation above, external replacement resorption can be classified as either: (a) external transient replacement resorption, or (b) external progressive replacement resorption.

Clinically, as a result of the ankylosis that occurs prior to external replacement resorption, teeth with this type of resorption will have reduced mobility and a different percussion sound and feeling. Some authors have described the sound as being “high‐pitched”, “metallic” or “woody” but it is variable according to the extent of the resorption. The key point is that the percussion sound is usually dull and very different to the adjacent teeth that do not have ankylosis and replacement resorption. The patient will typically have a history of a traumatic injury to the tooth and there are usually no symptoms reported by the patient. The tooth may appear submerged in advanced cases, particularly if the resorption commenced at a young age, such as prior to puberty.^46^, ^47^

Radiographically, there will be areas where external tooth substance (cementum and dentin) has been resorbed and bone has replaced it. The outline of the remaining root may be quite irregular as the resorption may be more rapid in some areas than others. There will be no PDL space and the lamina dura will be lost in areas where this resorption has occurred.^29^

Management of external progressive replacement resorption usually consists of monitoring the tooth on a regular basis with clinical review examinations and periapical radiographs to determine the rate of resorption and to prepare the patient for eventual loss of the tooth. In addition, the site should be considered for subsequent prosthetic replacement of the tooth, especially if it is an anterior tooth due to the aesthetic concerns involved. Consideration of the patient's age and facial growth stage is important because the resorbing tooth will not erupt any further and it will impede the alveolar downgrowth, resulting in a defect that is difficult to manage in the long‐term. Such cases may be suitable candidates for decoronation just below the cemento‐enamel junction and “root burial” to allow the alveolar bone to develop normally, while maintaining bone in the site as the tooth resorbs.^47^ External transient replacement resorption does not require any treatment but the tooth must be examined regularly before such a diagnosis can be determined.

Prevention of external replacement resorption is the best approach, where possible—but this is not always feasible as the PDL and root damage typically occurs before a person who has experienced trauma to a tooth sees a dentist. If a dentist is contacted after an injury such as an avulsion, then the dentist can provide advice regarding the first aid management of the tooth but even this may be too late. The most important preventive measures include reducing the extra‐oral time of avulsed teeth, encouraging the use of an appropriate storage medium if the tooth cannot be replanted, avoiding further damage to the PDL and root during the replantation or repositioning of the tooth by the dentist, using a functional (i.e. flexible) splint, avoiding extra‐oral root canal treatment and avoiding the immediate use of toxic root canal medicaments such as Ca(OH)_2_.^6^, ^21^, ^37^ It is important to also understand that external replacement resorption is not related to the dental pulp so root canal treatment will not prevent or arrest this type of resorption. However, many teeth that are susceptible to external replacement resorption (such as avulsed and intruded teeth) will also be likely to develop external inflammatory resorption and therefore root canal treatment may be necessary to prevent the inflammatory resorption, as discussed above.

### 6.4 External invasive resorption

External invasive resorption is an insidious process that is not fully understood, especially with respect to its aetiology and pathogenesis. It has been given numerous names in various publications^5^ and has been commonly called “cervical resorption”. However, it does not always occur in the cervical part of a tooth, especially if there has been gingival recession. Since the resorption is external in origin, it must always commence in a sub‐gingival location but it can then spread throughout the tooth in all directions—that is, horizontally, coronally and apically.^48^, ^49^ It can also develop in the crown of an unerupted tooth via an enamel defect.^50^, ^51^ Hence, even if it commences in the cervical region of the tooth root, it will spread to other parts of the tooth which implies that the term “cervical” is inappropriate. The term “invasive” is preferred as it describes the nature of the resorbing tissue and how it spreads throughout the tooth.

**FIGURE 10:**
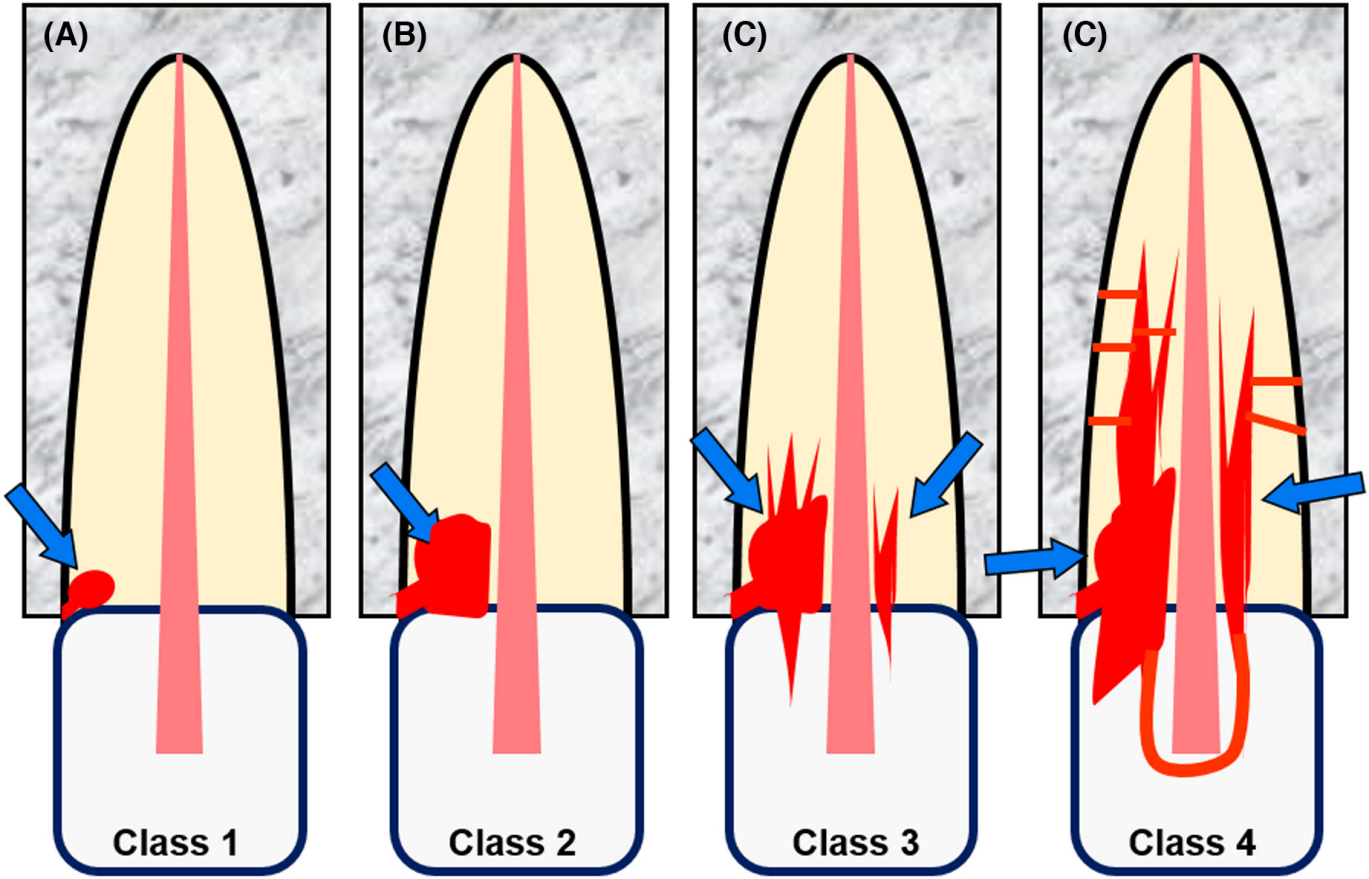
External Invasive Resorption. Schematic diagrams of four stages of this type of resorption, according to Heithersay (1999b). (A) Class 1; (B) Class 2; (C) Class 3; (D) Class 4

**FIGURE 11:**
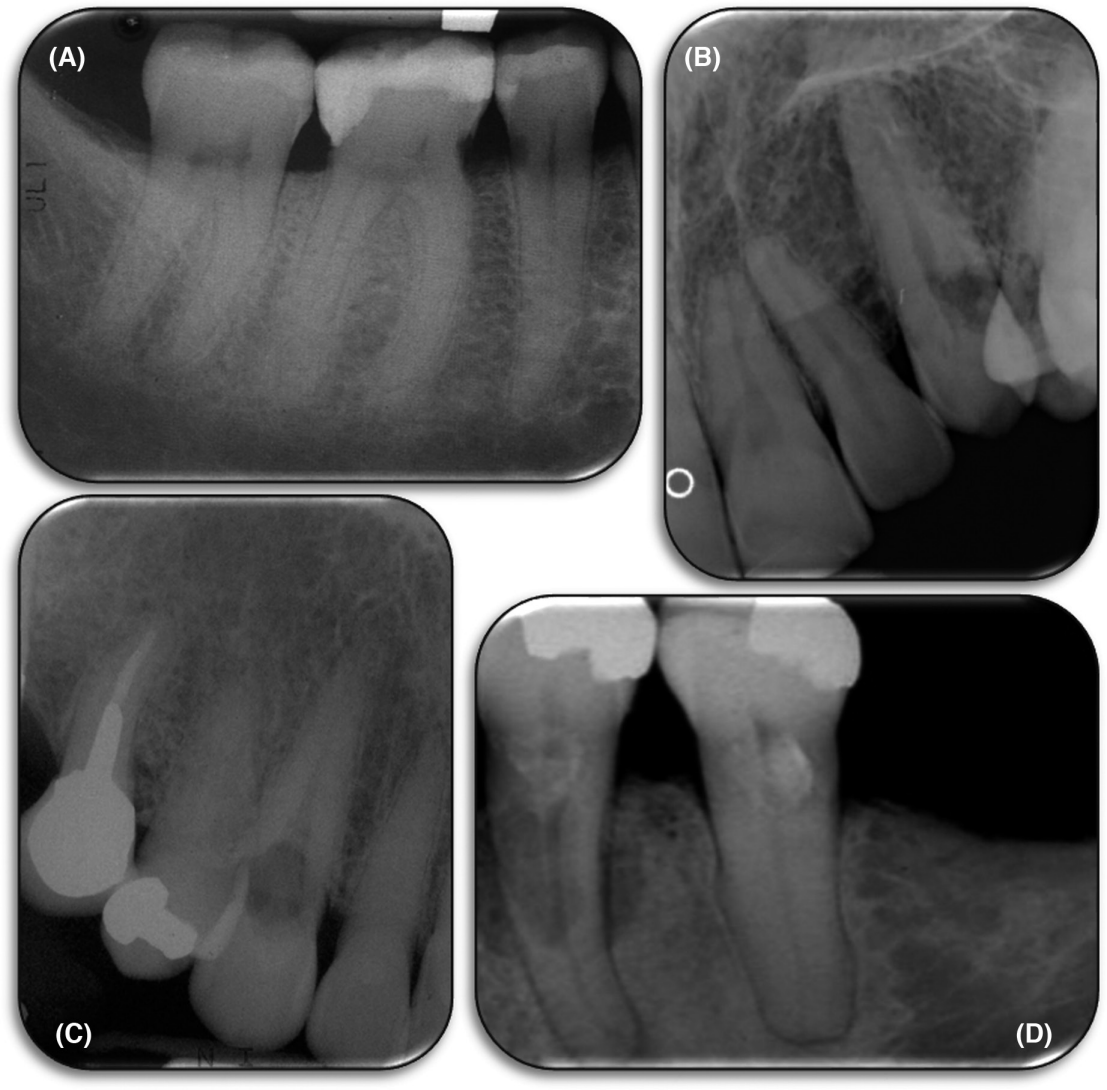
External Invasive Resorption. Periapical radiographs showing examples of the four stages of this type of resorption, according to Heithersay (1999b). (A) Class 1—mesial surface of tooth 36 and distal surface of tooth 35; (B) Class 2—distal and labial surfaces of tooth 23; (C) Class 3—palatal surface of tooth 13; (D) Class 4 mesial, buccal and lingual surfaces of tooth 34

Heithersay^48^, ^49^, ^52^, ^53^ has extensively investigated this type of resorption and he described it as a relatively uncommon, insidious and often aggressive form of external tooth resorption, which may occur in any tooth of the permanent dentition. Heithersay described this type of resorption as having four stages which he called Classes 1, 2, 3 and 4 (Figures 10 and 11).^52^ The Class is determined by the radiographic appearance of the resorptive defect on periapical radiographs. An alternative classification, based on three‐dimensional computerised tomography, was proposed by Patel et al.^54^—this can be useful to direct treatment decisions if such images are available. However, the Heithersay classification is generally easier to use and only requires a periapical radiograph which is usually the first image taken by practitioners to assess a tooth when this type of resorption is found in a tooth.

Initially, the invading tissue is a fibrous connective tissue that is very vascular—hence it bleeds profusely if probed. Later, as the resorption progresses, ectopic calcific, bone‐like tissue develops. Histologically, and sometimes radiographically, small “finger‐like” channels or projections can be seen extending further into the dentin and connecting with the PDL which then provides multiple entry points for blood vessels and the invading clastic cells.^42^, ^43^, ^44^, ^45^, ^48^, ^49^, ^52^, ^53^

External invasive resorption does not often have a clear or definite aetiology. There are various theories with the most likely seeming to be a developmental defect at the cemento‐enamel junction where the two tissues do not meet or do not overlap, leaving exposed dentin. Cementum may also be missing following trauma to the tooth, after repeated scaling and root planing procedures during periodontal treatment, after restorative dental procedures or after surgical procedures. In addition, some other predisposing factor or incident needs to be present or has to occur to stimulate the resorbing cells to invade the tooth. However, studies have not been able to prove this theory and it remains largely a mystery in many cases. Heithersay^52^ outlined a series of “potential predisposing factors” that he was able to ascertain from the histories of a series of patients—but there was no direct evidence to indicate that any of these factors actually caused the resorption; instead they were associated with the tooth in question so they may have had some influence rather than being a sole, direct or definite cause.^49^, ^52^

Clinically, patients with external invasive resorption usually do not have any symptoms and there may or may not be any clinical signs.^29^ The presence or absence of clinical signs will depend on the location and extent of the resorption, plus any concurrent pathosis (e.g. pulp disease). Sometimes patients will report a “different feeling” associated with the tooth such as when they brush their teeth. Some patients may notice bleeding of the gingiva whereas others may notice a pink discolouration of the tooth. Many cases are an incidental finding during a routine dental examination, during a scaling or root planing procedure, or when a radiograph is taken for some other purpose. The tooth will usually have a clinically normal pulp and clinically normal periapical tissues. The defect can sometimes be probed, in which case the adjacent tissue usually bleeds readily during probing.^29^ There may be ankylosis and reduced mobility in the more advanced Class 4 cases.^45^, ^48^, ^49^

Radiographically, the appearance of this resorption varies according to the stage of the resorptive process—that is, the extent of the defect created by the resorption. The defect is usually an irregular radiolucency within the tooth but there are many variations in appearance.^45^, ^48^, ^49^ As the defect becomes larger, it may appear to surround the pulp leaving the root canal walls intact. In the more advanced stages, the invading tissue spreads throughout the tooth in all directions as described above. As the ectopic bone‐like tissue forms,^42^, ^43^, ^44^, ^45^, ^48^, ^49^ the defect becomes a mixed radiolucent‐radiopaque area within the tooth where tooth structure has been resorbed, giving a somewhat “moth‐eaten” appearance.^19^ The radiographic appearance of external invasive resorption is very different when compared to all other forms of tooth resorption which is a feature that can be used to diagnose its presence. In other words, if the resorptive defect does not have the classic appearance of any other form of tooth resorption, then it is likely to be external invasive resorption.

The management of external invasive resorption depends on the stage of the resorptive process, which is largely dependent on the size of the defect. Heithersay reported a treatment approach that uses trichloroacetic acid to cauterize the resorbing (invading) tissue followed by curettage of the defect and then restoration with glass ionomer cement.^15^, ^49^, ^53^ A more comprehensive restoration may be required once the tooth has been reviewed to ensure the resorptive process has ceased. Heithersay reported excellent results when treating Class 1 and Class 2 invasive resorptive defects with this approach. The results for Class 3 defects were quite good but these teeth often needed other treatment such as root canal treatment, periodontal surgery for access or crown lengthening, orthodontic extrusion, crowns, etc. Some Class 3 cases were better managed via an internal approach where root canal treatment was also required, but with the advantage of not losing any more external tooth structure and maintaining the periodontal tissues—especially in anterior teeth with proximal resorptive defects where the interdental papilla may be at risk. The Class 4 cases did not have good or predictable outcomes with the above treatment approaches and therefore these teeth can simply be monitored with regular clinical and radiographic examinations. The resorptive process appears to be very slow or even inactive in the advanced Class 4 cases so the tooth may be kept until problems develop. Such patients need to be made aware of the possible problems that can develop—such as root fracture, pulp disease, periodontal disease, ankylosis, etc.—and when any of these occur, the tooth should be extracted.^15^, ^49^, ^53^

### 6.5 External pressure resorption

**FIGURE 12:**
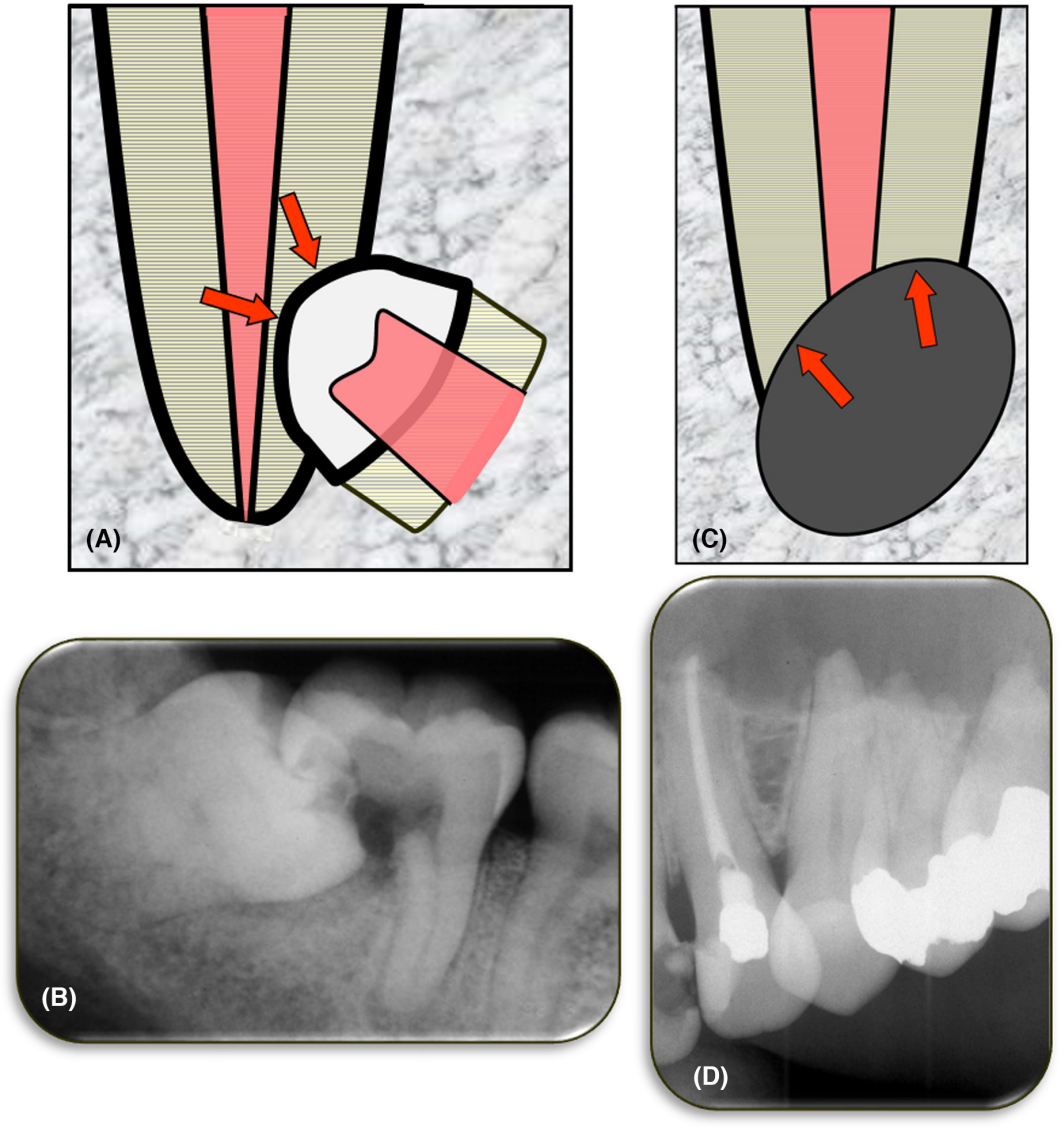
External Pressure Resorption. (A) Schematic diagram of external pressure resorption caused by an impacted tooth located adjacent to the tooth undergoing resorption; (B) Periapical radiograph of tooth 47 with extensive pressure resorption caused by the mesially‐angled impacted tooth 48; (C) Schematic diagram of external pressure resorption caused by a tumor, cyst or other pathological condition located adjacent to the tooth undergoing resorption; (D) Periapical radiograph showing pressure resorption of the apices of teeth 23, 24, 25 and 26 which was caused by an odontogenic keratocyst in the left maxilla

External pressure resorption is the resorptive process that occurs when there is pressure applied to the external surface of a tooth root (Figure 12). The term “pressure” is used because it indicates that the resorption is due to the pressure being applied to the tooth.^55^

There are two situations where pressure resorption can occur and they are dependent on what is causing the pressure. Firstly, the pressure on a tooth root may occur when there is an adjacent impacted tooth (Figure 12A,B) or when there is a tooth with an aberrant eruption pathway. Secondly, the pressure may be caused by a pathological condition such as a tumor or cyst that is expanding and placing pressure on the tooth (Figure 12C,D). Slowly expanding tumors or cysts (such as an ameloblastoma, giant cell tumor and fibro‐osseous lesions) are more likely to cause pressure resorption of an adjacent tooth than a rapidly expanding lesion, where the latter is more likely to displace the tooth roots.^32^

Clinically, there are usually no symptoms associated with the tooth that is undergoing the pressure resorption, especially in the early stages of the process.^29^ In the later stages, there may be symptoms associated with the pulp if the resorption has progressed to involve the pulp and the root canal system. However, the patient may have symptoms arising from the impacted tooth, or from the tumour or cyst that is causing the resorption. The exact nature of such symptoms will vary according to the individual situation (such as type of impaction, degree of impaction, type of tumour, type of cyst, location, size, etc.).^29^

The resorbing tooth will have a clinically normal pulp and clinically normal periapical tissues, unless there is concurrent pulp disease as a result of other causes (such as caries, restoration breakdown, cracks, etc.). As the resorption progresses, the tooth may have increased mobility and the patient may experience tenderness to biting. The tooth may have a “different” percussion sound and feeling compared to other teeth that are not resorbing. The tooth is not normally ankylosed.^29^

Radiographically, there will be loss of tooth structure with an adjacent impacted tooth impinging on the tooth undergoing resorption. If the resorption is due to an aberrant pathway of eruption of an adjacent tooth, then that tooth may still be impinging on the tooth undergoing resorption or it may have erupted and is no longer causing resorption. If the pressure on the resorbing tooth is from an adjacent cyst or tumour, then there will be a radiolucency, a radiopacity, or a mixed radiolucent‐radiopaque area adjacent to the site of the resorption.^29^

The management of external pressure resorption depends on the cause of the pressure and the extent of the resorption. If the resorption is caused by an impacted tooth then either the impacted tooth or the tooth that is resorbing should be extracted. In some cases, both teeth will need extraction. If the resorption is caused by an aberrant eruption pathway of an adjacent tooth, then the tooth causing the resorption could be extracted or orthodontically moved to avoid the resorbing tooth. In some cases, the resorbing tooth may need to be extracted. If the tooth that had the aberrant pathway has fully erupted, then the tooth with the resorption may be able to be monitored over time, or it may need extraction, depending on the extent of the resorption. If the pressure resorption is caused by a cyst or tumour, then the lesion should be surgically removed and managed according to the specific diagnosis of the condition. The resorbing tooth may also need to be extracted as part of the treatment of a cyst or tumour, depending on the extent of the resorption, its location, etc. If the resorbing tooth is not removed as part of the treatment of the cyst or tumour, then it will need to be monitored post‐operatively to detemine whether the resorption continues and whether there are any other complication such as pulp necrosis and infection.

### 6.6 Orthodontic resorption

**FIGURE 13:**
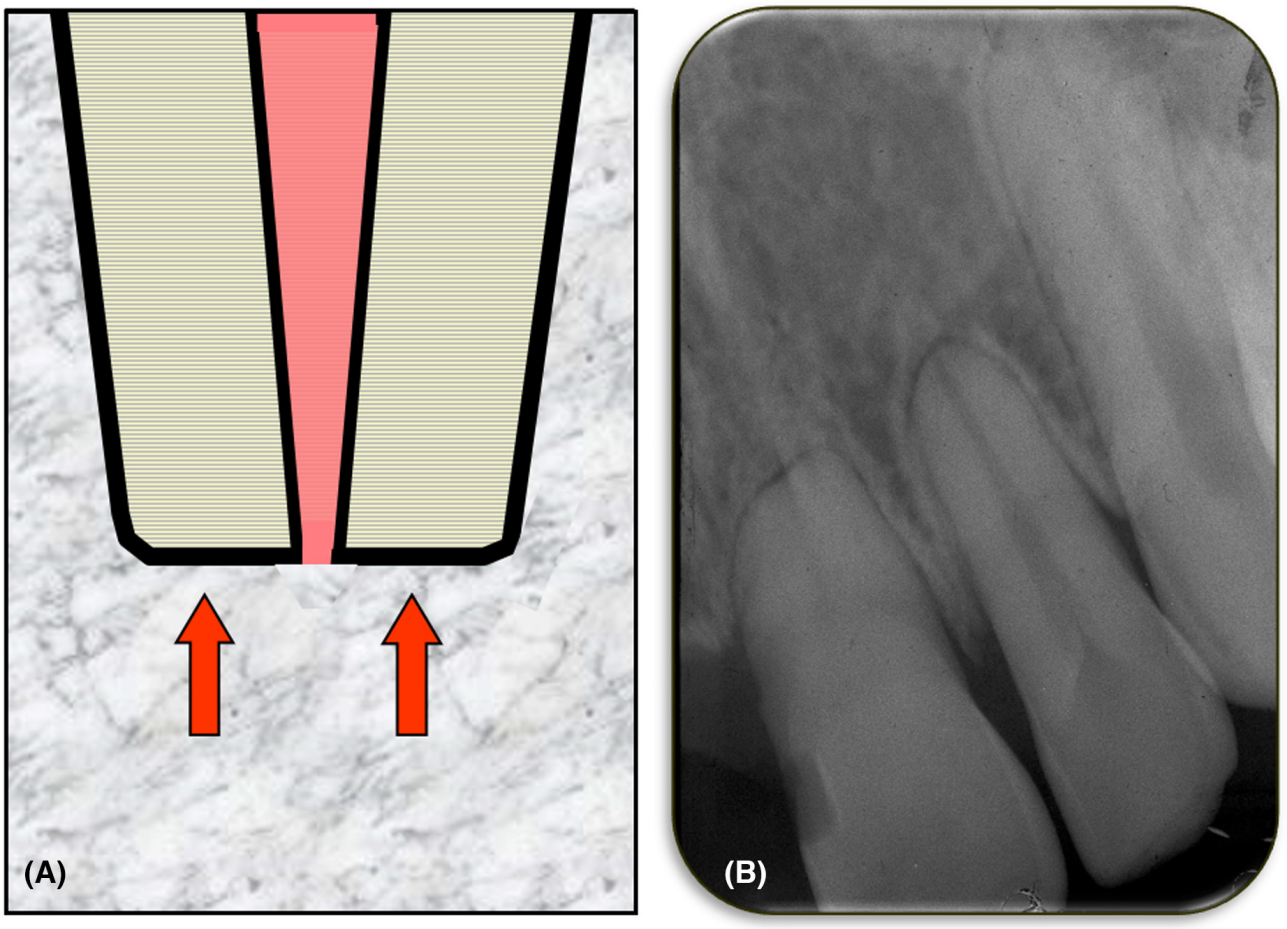
Orthodontic Resorption. (A) Schematic diagram; (B) Periapical radiograph of teeth 21 and 22 showing orthodontic resorption. The patient reported having fixed appliance orthodontic treatment for about 18 months when he was a teenager. Both teeth had increased mobility

Orthodontic resorption is the process by which the apical part of one or more teeth undergo resorption, resulting in shortened roots (Figure 13). The term “orthodontic” is used because the resorption occurs during orthodontic treatment and there is no other aetiology. In some ways, this resorption could be considered to be similar to “pressure resorption” but there are two distinct differences. The first difference is the specific aetiology—that is, the forces that are generated during orthodontic treatment whereas, in contrast, pressure resorption is the result of pressure from an impacted tooth or an adjacent pathological condition such as a tumour or cyst (see above). The second difference is the site of the resorption which is always apical whereas pressure resorption can occur anywhere along the length of the tooth root. Hence, the more specific term of “external orthodontic resorption” is appropriate.

Clinically, orthodontic resorption usually has no symptoms or signs unless it has progressed to a very advanced stage where the tooth becomes very mobile.^29^ The clinical manifestations of orthodontic root resorption are often masked by the splinting effect of the orthodontic arch wires and the normal discomfort associated with orthodontic treatment. It is also likely that clinicians expect a certain degree of increased mobility during active orthodontic treatment so the increased mobility may go unnoticed. There may be early signs of orthodontic resorption if there is concurrent pulp pathosis as a result of an incidental factor such as trauma to the tooth. Orthodontic resorption itself does not cause pulp pathosis. The diagnosis of orthodontic resorption is often only made when a radiograph is taken during orthodontic treatment to assess progress of such treatment, or when post‐treatment radiographs are taken. Radiographs may also be taken when there is increased mobility and tenderness during orthodontic treatment or when persistent mobility is noted after removing the orthodontic appliances.

Radiographically, the roots appear shortened and blunted with rounded apices. The lamina dura usually appears normal and the PDL space may be normal or it may be widened as a result of the orthodontic tooth movement rather than because of the resorption. There will be no signs of ankylosis and no periapical radiolucency or radiopacity. The apical foramen is typically “open” (i.e. lacking an apical constriction) because of the apical shortening of the tooth root.^29^ The apical foramen may subsequently become narrower as the pulp produces secondary dentin as part of its normal physiological processes as the patient becomes older.

Management depends on when the resorption is diagnosed. If it is diagnosed during orthodontic treatment, then the orthodontic treatment can be stopped or the forces applied to the involved teeth can be reduced. However, reduction of forces is not guaranteed to stop the resorption. Consideration of whether to cease the orthodontic treatment will require careful consideration as the teeth may not be in a stable occlusal relationship or they may not be in an aesthetic position. A modified course of treatment with compromised, altered or reduced expectations may be necessary.

If orthodontic resorption is diagnosed after the orthodontic treatment has been completed (e.g. on post‐treatment radiographs), then the resorption should not continue since the orthodontic forces are no longer being applied to the tooth/teeth, However, regular monitoring of the tooth/teeth with further radiographs (such as, after 6 months) should be arranged to ensure the resorption has stopped, as well as to assess whether the mobility has reduced, the pulp, periapical and periodontal tissues are healthy, and the patient is maintaining a high level of oral hygiene.

The prognosis of teeth with orthodontic resorption depends largely on how much resorption has occurred—that is, how short the tooth roots have become. Patients who have teeth with orthodontic resorption should be advised to maintain excellent oral hygiene to prevent periodontal disease as teeth with shortened roots are at a higher risk of problems (such as the need for extraction) if periodontal disease develops in the future. Patients should also be counselled to use a mouthguard when participating in sports or when involved in any activities where trauma to the teeth is a risk. The use of a mouthguard is particularly important because the teeth most likely to undergo traumatic injuries are the maxillary central incisors, followed by the maxillary lateral incisors and mandibular incisors^56^, ^57^ and these same teeth have been reported to be the most common teeth to have orthodontic resorption.^58^, ^59^

### 6.7 Physiological resorption

**FIGURE 14:**
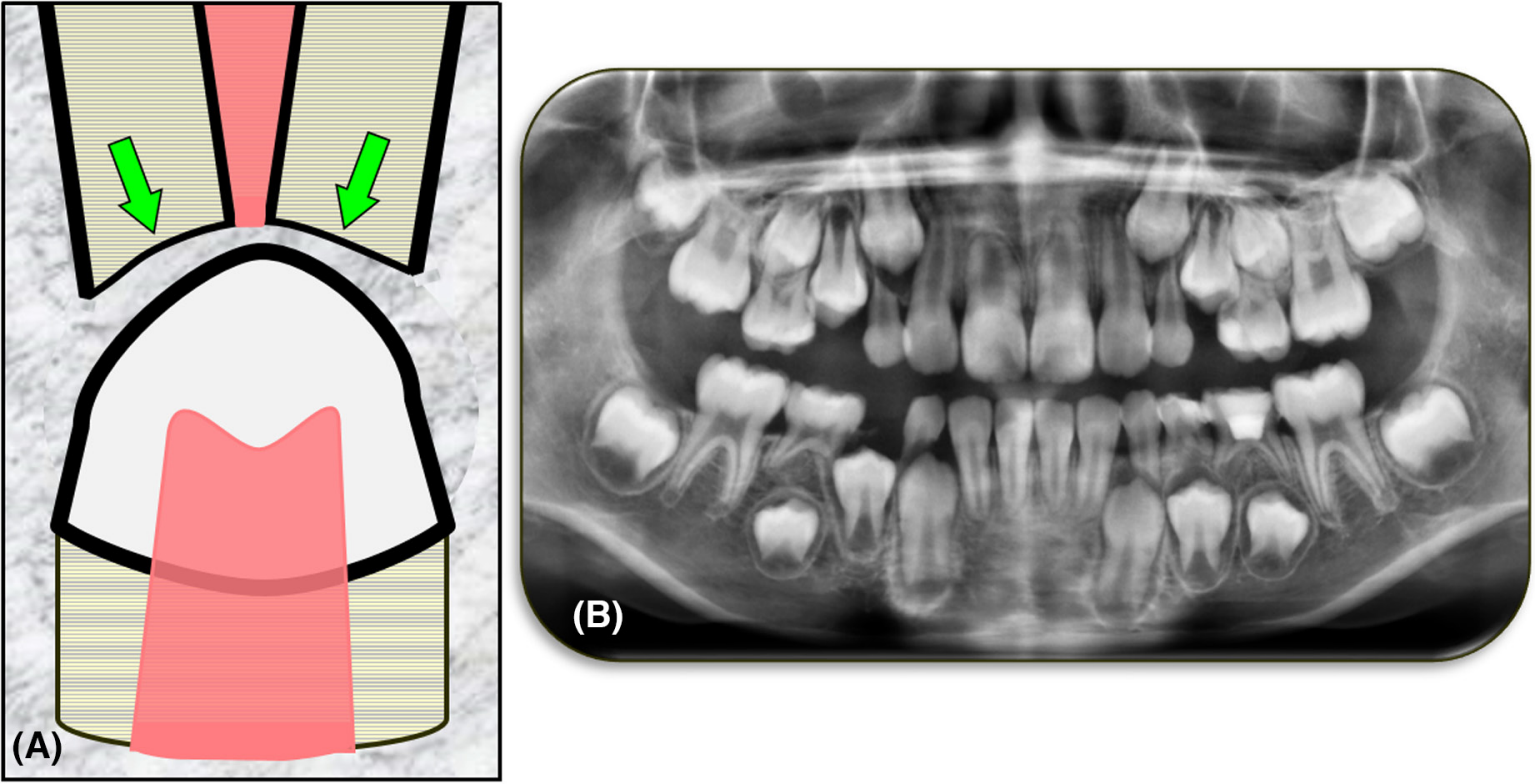
Physiological Resorption. (A) Schematic diagram; (B) Cropped panoramic radiograph showing physiological resorption of multiple primary teeth—teeth 53, 55, 63, 65, 73, 74, 75, 83 and 85

Physiological resorption is the resorptive process that primary (deciduous) teeth undergo as they exfoliate (Figure 14). It is a physiological process, hence the use of the term “physiological”.

Physiological resorption of a primary tooth occurs as the permanent successor tooth develops and erupts. The primary tooth roots are usually resorbed from the apical end towards the crown of the tooth but in some cases the resorption may commence on the lateral aspect of the root, depending on the position of the erupting tooth relative to the primary tooth roots. Primary teeth without successor permanent teeth may also undergo physiological resorption although this tends to occur at a later age and at a slower rate. Some primary teeth without successors may not resorb at all, or to no significant extent.

Clinically, there may be a sense of pressure on the deciduous tooth with periods of intermittent increased mobility followed by firming up of the affected tooth. As the resorption proceeds, the increased mobility becomes more persistent due to the unfavorable crown to root ratio that develops. Discomfort with mastication is a common finding. Just before exfoliation, the crown of the primary tooth may appear pink in color, often with only a thin enamel shell remaining which encases well‐vascularized granulation tissue.^29^ During the resorptive process, the pulp in the crown of the primary tooth remains unaffected, unless there is concurrent caries or breakdown of any restorations. The resorption continues intermittently until the whole or greater part of the root(s) has/have been resorbed and the tooth exfoliates.

Radiographically, loss of tooth substance from the primary tooth roots will be evident, thus the roots become shorter and thinner. If there is a permanent successor tooth, then this will be seen under the resorbing roots of the primary tooth.^29^ The morphology of the resorbing roots depends on the eruptive pathway of the permanent tooth. For example, mandibular incisors typically erupt from the lingual side while maxillary lateral incisors erupt from the palatal side of their primary tooth counterparts and therefore the primary teeth will usually resorb initially on their lingual/palatal aspects.

Management of physiological resorption is limited to either monitoring the exfoliation process or extraction of the primary tooth. Extraction is required if the primary tooth has ankylosed, has ceased or delayed resorption, the tooth is interfering with the eruption of the permanent tooth, the tooth is causing discomfort (usually associated with chewing which may also affect the child's diet), the tooth or surrounding tissue becomes infected, or the tooth does not exfoliate because of the persistence of coronal PDL fibres.

### 6.8 Idiopathic resorption

**FIGURE 15:**
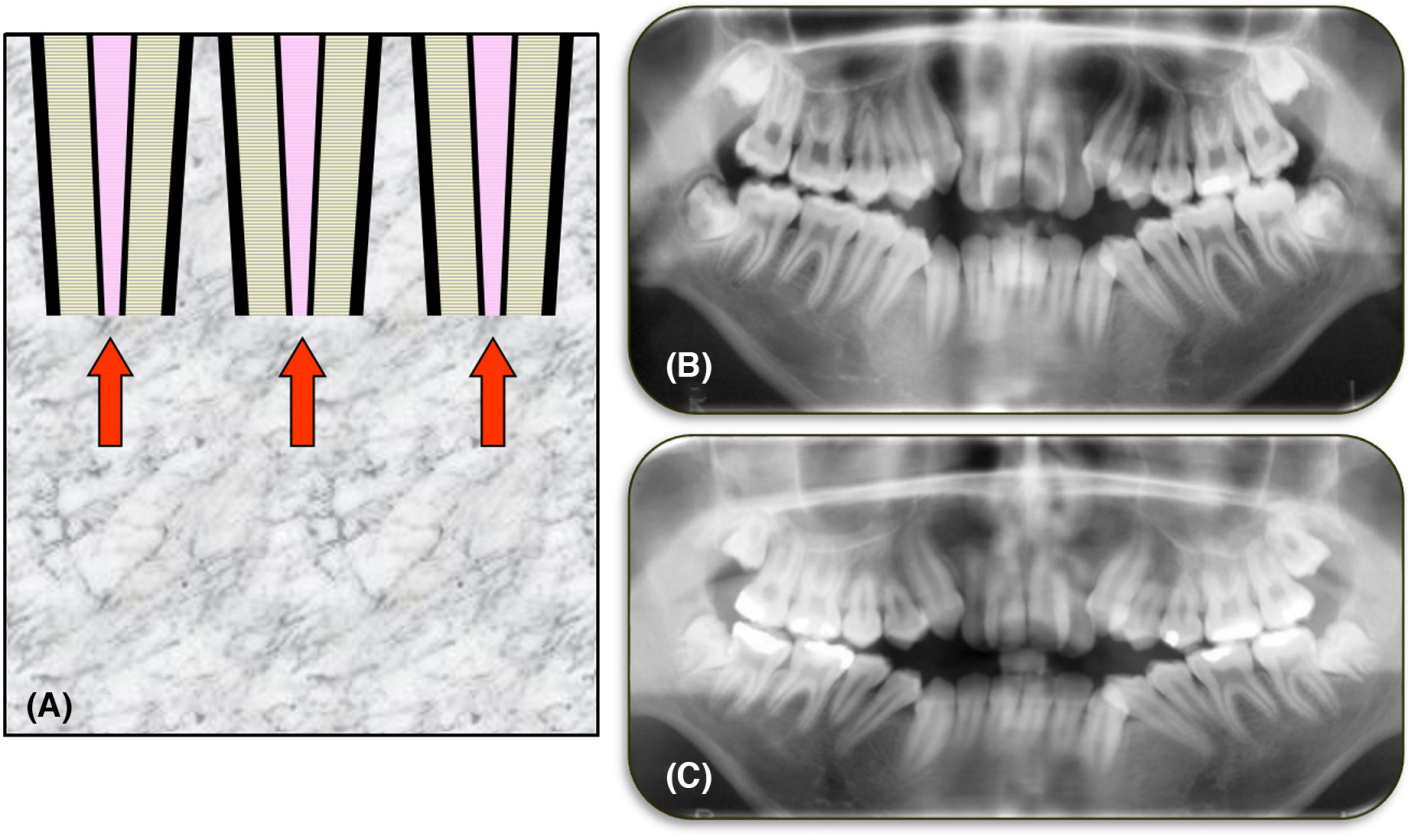
Idiopathic Resorption. (A) Schematic diagram of multiple teeth with idiopathic resorption; (B) Cropped panoramic radiograph of a male patient who had an open bite associated with the anterior and premolar teeth; (C) Another panoramic radiograph of the same patient taken 18 months later which suggests that the maxillary premolars and the mandibular second premolars have had idiopathic resorption resulting in shortened roots. When the lengths of other teeth are compared, there is very little difference between the two images apart from what may be expected as a result of the slightly different angulations of the patient, sensor, etc. when the panoramic radiographs were taken. In contrast, the maxillary premolars and mandibular second premolars are much shorter, suggesting idiopathic resorption has occurred. The patient did not have any orthodontic or other treatment during the 18‐month period between the radiographs and he had no systemic diseases/conditions

Idiopathic resorption is defined as tooth resorption with no apparent cause—thus the use of the term “idiopathic”. This type of resorption has been recognised for many years with Shafer et al.^60^ reporting that many permanent teeth may undergo some resorption without any obvious cause. Fortunately, this type of resorption is rare and in most cases it is mild with only a small amount of tooth structure being lost – typically less than 4 mm.^60^, ^61^ Typically, this type of resorption involves multiple teeth which have shortened roots (Figure 15) compared to the patient's other teeth. They are also shorter than what would normally be expected for the specific tooth types involved.

Teeth with idiopathic resorption have no history of trauma, orthodontic treatment, radiation treatment or any other obvious cause of the resorption. Multiple teeth are usually involved. In most cases, the patient's medical history is unremarkable but there may be some possible systemic associations such as hypoparathyroidism, pseudohypoparathyroidism, Gaucher's Disease, Turner's Syndrome, malnutrition and Paget's Disease. Resorption associated with systemic disorders usually affects multiple teeth and is bilaterally distributed.^60^ Idiopathic resorption usually involves the apical parts of the tooth roots, resulting in shortened roots. This type of resorption should be distinguished from that reported by several authors many years ago where the term “idiopathic resorption” was used to describe cases where there were multiple teeth with external invasive resorption—some examples are the reports by Thoma et al.,^62^ Stafne et al.^63^ and Kerr et al.^64^

Clinically, there are no symptoms or signs associated with teeth that have had, or are currently undergoing, idiopathic resorption.^29^ The resorption is usually an incidental finding seen when radiographs have been taken for some other clinical reason. The teeth have clinically normal pulps and clinically normal periapical tissues along with normal periodontal tissues, unless there is concurrent pulp disease as a result of other causes—such as caries, restoration breakdown, cracks, etc.^29^ or concurrent periodontal disease.

Radiographically, the roots appear shortened with rounded or somewhat flattened apices, similar to that seen with orthodontic resorption. The PDL space and lamina dura usually appear normal—that is, there is no widening of the PDL space, no loss of lamina dura and no ankylosis.^29^

Management includes checking for any possible systemic causes—e.g., via blood tests. If there are no systemic causes, then the progress of the resorption should be monitored both clinically and radiographically. There is no interceptive treatment indicated, or possible, particularly because the aetiology of the resorption is unknown.

## 7 DISCUSSION

There are 11 different types of tooth resorption (Table 1). Each type has its own aetiology, pathogenesis, clinical and radiographic features. Each type requires specific management strategies which can range from simple regular observation and monitoring through to complex treatments such as root canal treatment, extraction and various surgical procedures. Each type of resorption is a complex phenomenon that can have serious consequences for the tooth and surrounding tissues. The consequences of resorption can also be very complicated for the patient, especially if one or more teeth need to be extracted and the resultant site needs prosthetic management.

It is essential that dentists understand the different types of resorption along with their aetiology and pathogenesis so they can diagnose the resorption correctly and appropriately advise the patient regarding the management of the specific tooth.

The first step towards understanding and making the correct diagnosis of any disease or physiological condition is to have a good classification of the different diseases and/or conditions. As discussed above, there have been at least 16 attempts over many years by authors to develop a classification for the many types of tooth resorption. However, unfortunately, most of these classifications have not been ideal with inconsistent approaches that were most commonly based on aetiology rather than on the anatomy, physiology and/or pathology of the resorption.

The classification of tooth resorption presented above follows the recommended approaches for developing disease classifications in that it is possible to use, meaningful, useful, clear and universal.^7^, ^10^ It enables easy storage, retrieval and analysis of health information for evidenced‐based decision‐making, it can be shared and health information can be compared between institutions, different settings and countries.^11^ A combination of anatomical, physiological, pathological and aetiological approaches have been used to develop this classification.^12^

Clinicians and researchers are encouraged to adopt this classification of tooth resorption for their clinical practice, research and publications. This will result in a universal approach to the diagnosis and management of resorptive conditions in the dental profession. In addition, it will facilitate clear communication about tooth resorption within the profession which will ultimately be of benefit to patients.

## 8 CONCLUSION

Tooth resorption is either a physiological or a pathological process that results in loss of dentin and/or cementum. It may also be associated with loss of bone. A classification of tooth resorption has been proposed that uses simple, relevant and appropriate terminology based on the nature and location of the resorptive process that occurs in the tooth. There are two broad categories of internal and external tooth resorption which are then sub‐divided into three types of internal tooth resorption (surface, inflammatory and replacement) and eight types of external tooth resorption (surface, inflammatory, replacement, invasive, pressure, orthodontic, physiological and idiopathic). The understanding, diagnosis and management of tooth resorptive processes can be facilitated by using this simple classification. Ideally, this classification should be used universally by the entire dental profession to ensure clarity and to avoid confusion.

## AUTHOR CONTRIBUTIONS

The authors contributed equally to the development of this classification and the writing of he article.

## CONFLICT OF INTEREST

Paul Abbott occasionally acts as a Consultant and/or Lecturer for OzDent Pty Ltd for which he is paid an Honorarium. Shaul Lin declares that he has no conflicts of interest with this work.

## Data Availability

Data sharing not applicable to this article as no datasets were generated or analysed during the current study.
